# Method Improving for Isolation and Characterization of Allergy-Inducing Polymer Impurities in Cefotaxime Sodium Medicines Available From Pharmaceutical Industry

**DOI:** 10.3389/fchem.2022.820730

**Published:** 2022-02-28

**Authors:** Yanan Fu, Jin Li, Fang Feng, Lihui Yin

**Affiliations:** ^1^ Division of Antibiotics, Institute for Chemical Drug Control, National Institutes for Food and Drug Control, Beijing, China; ^2^ Department of Pharmacy, China Pharmaceutical University, Nanjing, China

**Keywords:** cefotaxime sodium, polymer, impurity, dimer, trimer, nuclear magnetic resonance

## Abstract

**Objective:** To prepare and validate the chemical structure of the cefotaxime dimer and cefotaxime trimer impurities available from pharmaceutical industry.

**Methods:** A polymer stock solution of cefotaxime sodium was obtained using a concentrated solution degradation method. The cefotaxime dimer and trimer impurities were separated and purified by preparative reversed-high-performance liquid chromatograph (RP-HPLC), and the pure cefotaxime dimer and trimer were obtained by a freeze-drying method. The two impurities were characterized by infrared (IR) spectroscopy, ultraviolet (UV) spectroscopy, mass spectroscopy, and nuclear magnetic resonance (NMR, including one-dimensional and two-dimensional NMR). The groups corresponding to the characteristic absorption peaks in the UV and IR spectra were analyzed, and the ^1^H and ^13^C NMR signals were assigned.

**Results:** The cefotaxime dimer was isolated and purified from the actual sample of industrial medicines, and chemical structure of the dimer is the same as in the dimers investigated earlier. The polymerization sites and stereoscopic configuration of trimer impurity was validated for the first time.

**Conclusion:** This study is of great significance for the study of the structures and quality control of polymer impurities in cephalosporin drugs. Promoting the polymerization sites study and providing a technical basis for allergic study of polymer impurities.

## 1 Introduction

β-lactam antibiotics have been widely used in clinical practice because of their unique advantages in anti-infective treatment. However, the raw materials and preparation process of β-lactam antibiotics may result in polymer impurities during production, transportation, storage, and even use. Polymer impurities, mainly including dimers, trimers, tetramer, and so on, are generated through covalent polymerization between β-lactam antibiotic drug molecules. β-lactam antibiotic molecules typically contain free polar groups such as amino and carboxyl groups, as well as structurally unstable quaternary lactam rings and easy-to-fall-off three-digit side chain groups. As a result, they are prone to polar groups attacking the amide bond of the quaternary lactam ring, resulting in fully polymerized polymer impurities, as well as attacking the methylene group of three-digit side chains, resulting in incompletely polymerized polymer impurities that fall off three-digit side chains, and a few polymer impurities with an unclear connection sites. These can be as the multivalent haptens, which are associated with allergic reactions and can seriously threaten people’s health (Buonomo et al., 2016; Hu, 2020; Department of Medical Administration, 2021). Therefore, the control and research of polymer impurities are particularly important. At present, with the rapid development of analytical technology, the analysis methods of cephalosporin antibiotic polymer impurities have been expanded from the earliest G10 chromatography (Sephadex G10) to high-performance size exclusion chromatography (HPSEC), reversed-phase high performance liquid chromatography (RP-HPLC), high-performance capillary electrophoresis (HPCE), two-dimensional chromatography (2D-HPLC), and liquid chromatography–mass spectrometry (LC-MS), and various technologies have been used to control the polymer impurities in β-lactam antibiotics (Hu, 2008; Li et al., 2013; Wang et al., 2015). The specificity and separation ability of analytical methods have been improved to varying degrees. Nevertheless, there are still certain challenges in the study of the structures of polymer impurities and the polymerization sites. Mass spectrometry can only be used to preliminarily infer the planar structures of polymer impurities based on the relative molecular masses of impurities, fragment ions, and cleavage rules (Qian et al., 2017; Xu et al., 2018; Wang et al., 2019; Hu et al., 2020; Chong et al., 2021; Li et al., 2021a; Li et al., 2021b; Li et al., 2021c; Zhang et al., 2021), but it cannot confirm the exact structures, polymerization sites, and stereoscopic configurations of polymer impurities in cephalosporin antibiotics.

Cefotaxime sodium is a third-generation, semi-synthetic cephalosporin developed by Sanofi-Aventis. It has a broad antibacterial spectrum and strong antibacterial activity. It has been mainly used in clinics to treat respiratory symptoms caused by sensitive bacteria as well as infections of the respiratory system, urinary system, skin, and soft tissues, and it has had significant effects. Based on literature research, the structure of the cefotaxime dimer (Agarwal et al., 2004) was investigated and published by PubChem (NIH) (https://pubmed.ncbi.nlm.nih.gov). ChP 2020 (Chinese Pharmacopoeia Commission, 2020) specifies that G10 chromatography should be used to control the quality of polymer impurities. JP17 (Japan Pharmacopoeia Committee, 2016) also includes this drug, but the control of polymer impurities is not specified. USP 42 (United States Pharmacopeial Convention Inc, 2020) and EP 10.0 (European Directorate for the Quality of Medicine and Health Care, 2020) both include the cefotaxime dimer impurity (impurity F), but there have been no relevant reports confirming the structure of this polymer impurity, which is from the actual sample of industrial medicines. The trimer impurity has not been included in any pharmacopoeia. A method for the content determination of the polymer in cefotaxime by HPSEC has been reported (Dong et al., 2015). In a previous study, we also established a RP-HPLC method to control the dimer and trimer impurities in cefotaxime sodium and preliminarily estimated the dimer and trimer structures by a column switching LC-MS method (Li et al., 2020). However, due to the shortcomings of the LC-MS method, the connection sites and absolute configuration could not be confirmed.

**FIGURE 1:**
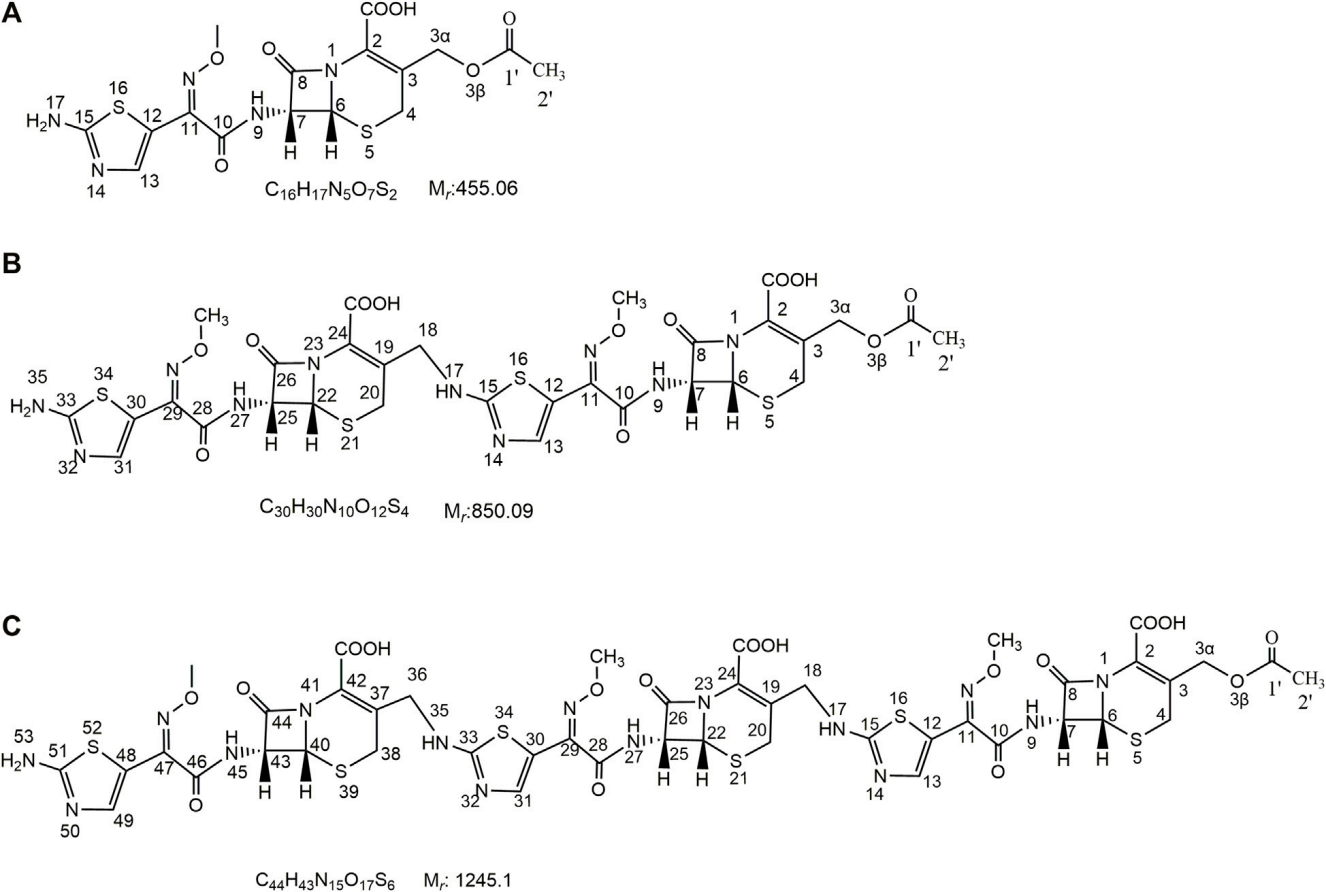
Chemical structures of cefotaxime, cefotaxime dimer and trimer.

In this study, we first obtained dimer and trimer stock solutions by a concentrated solution degradation method. Second, the dimer and trimer impurities (purity >90%) were obtained by a preparative chromatography and freeze-drying method. Finally, using infrared (IR) absorption spectroscopy, ultraviolet (UV) absorption spectroscopy, mass spectrometry (MS), and nuclear magnetic resonance spectroscopy (NMR) methods, the chemical structures and connection sites of the dimer and trimer were studied by comparison with the spectra of cefotaxime sodium (Sha et al., 2007; Anacona and Osorio, 2008). The chemical structures of cefotaxime sodium, its dimer, and its trimer are shown in Figure 1.

## 2 Materials and Methods

### 2.1 Instruments

The UV visible spectrophotometer was a SHIMADZU UV-2600 (Shimadzu, Chiyoda-ku, Tokyo, Japan). The IR spectrometer was a PerkinElmer Spott40 0FT-IR/NIR (PerkinElmer, Wiesbaden, Germany). The NMR spectrometer was a Bruker Avance 500 (Bruker, Zurich, Switzerland). The high-resolution mass spectrometer was a Q Exactive Focus (Thermo Fisher Scientific, Waltham, MA, United States). The software was Xcalibur 4.0. The preparative RP-HPLC system 2545B consisted of a 2545 binary pump, a 2998 photodiode array (PDA) detector, an SFO column oven, and a 2767 autosampler and fraction collector (Waters Corporation, Milford, MA, United States). The software was MassLynx V4.1. The rotary evaporator was a Hei-VAP Advantage ML/HB/G3 (Heidolph, Schwabach, Germany). The freeze-dryer was an Alpha 2-4 LSC Basic (Marin Christ, Osterode, Germany).

### 2.2 Samples and Reagents

HPLC-grade acetonitrile was purchased from Thermo Fisher Scientific (Waltham, MA, United States). Glacial acetic acid (98.0%) was supplied by Sigma-Aldrich Co. Ltd (St. Louis, United States), and dimethyl sulfoxide-*d*6 (DMSO-*d*6) was purchased from Beijing Chemical Works (Beijing, China). A milli-Q water purification system (Millipore, Bedford, MA, United States) was used to further purify the distilled water. Cefotaxime sodium, as a raw material, was provided by the National Institutes for Food and Drug Control of the People’s Republic of China. The purities of the cefotaxime dimer and trimer were >90.0%, as determined by HPLC, which were suitable for structural identification.

### 2.3 Reversed-Phase High-Pressure Liquid Chromatography Method

Mobile phase A was 0.05 mol/L phosphate buffer (pH 6.25) and methanol (86:14, v/v). Mobile phase B was 0.05 mol/L phosphate buffer (pH 6.25) and methanol (60:40, v/v). The gradient elution procedure was as follows: the proportion of phase B was maintained at 6.0% from 0 to 15.0 min, at 20% from 15.0 to 23.0 min, at 55% from 23.0 to 40.0 min, and at 100% from 40.0 to 54.0 min. Next, the proportion of phase B was maintained at 6% from 54.0 to 55.0 min and that of phase A was maintained at 6% from 55.0 to 65.0 min. The column was an Zorbax SB-C18 (4.6 mm × 150 mm, 5 μm) (Agilent Technologies, Santa Clara, CA, United States), the flow rate was 1.0 ml min^−1^, the column temperature was 30°C, the detection wavelength was 235 nm, and the injection volume was 20 µl.

### 2.4 Preparation Method

#### 2.4.1 Preparative High-Performance Size Exclusion Chromatography Method

Mobile phase A was 0.005 mol/L phosphate buffer (pH 7.0; a 0.005 mol/L disodium hydrogen phosphate solution was prepared, and the pH was adjusted to 7.0 with phosphoric acid). Mobile phase B was an acetonitrile. An isocratic elution was performed with A:B (v/v) = 95:5. The semi-preparative column was a TSK-Gel G2000SW (21.5-mm inner diameter (I.D.) × 30 cm, 13 μm), the flow rate was 5.0 ml min^−1^, the column temperature was 30°C, the detection wavelength was 254 nm, the injection volume was 1,000 μl, the fraction collecting time ranges were 11.3–12.0 min (fractions containing trimer impurities) and 12.2–13.1 min (fractions containing dimer impurities). The sample solution was a polymer stock solution.

#### 2.4.2 Preparative RP-HPLC Method

Mobile phase A was water and acetic acid (100:1, v/v). Mobile phase B was acetonitrile. A gradient elution was performed as follows: the proportion of phase B was 10.0% at 0.0 min, 15% from 10.0 to 15.0 min, 18% from 15.0 to 30.0 min, 30% from 30.0 to 42.0 min, 40% from 42.0 to 45.0 min, 45% from 45.0 to 50.0 min, 10% from 50.0 to 51.0 min, and 10% from 51.0 to 55.0 min. The semi-preparative column was a CAPCELL PAK CA18 MGII (10-mm I.D. × 25 cm, 10 μm), the flow rate was 5.0 ml min^−1^, the column temperature was 30°C, the detection wavelength was 235 nm, the injection volume was 1,000 μl, the fraction collecting time ranges were 33.4–35.3 min for the cefotaxime dimer and 38.0–39.9 min for the cefotaxime trimer, and the solutions were cefotaxime dimer and cefotaxime trimer stock solutions.

### 2.5 Ultraviolet Spectroscopy Method

First, 2, 2, and 1.5 mg of cefotaxime, cefotaxime dimer, and cefotaxime trimer were accurately weighed, respectively. The measured samples were then transferred into 100-, 50-, and 25-ml volumetric flasks, respectively. And the samples were dissolved and diluted with distilled water. The solutions were then shaken well, and the UV spectra in the wavelength range of 200–350 nm were obtained.

### 2.6 Infrared Spectroscopic Method

Approximately 1.0 mg each of cefotaxime, cefotaxime dimer, and trimer were accurately weighed. Total reflectance infrared spectroscopy (ATR) was conducted in the wavenumber range of 600–4,000 cm^−1^.

### 2.7 High-Resolution Mass Spectroscopy Method

High-resolution mass spectroscopy (HR-MS) was performed by direct injection at a rate of 10 μl·min^−1^. The scanning voltage (+) was 3000 V, the capillary temperature (+) was 350°C, the sheath gas (+) was 35 Lh^−1^, the auxiliary gas (+) was 10.00 Lh^−1^, the maximum spray current (+) was 100.00, the probe heater temperature (+) 350°C, the S-RF level was 50.00, and the ion source was heated electrospray ionization (HESI). A full-scan MS experiment (MS1) was conducted in the positive ion mode with a resolution of 70,000 and a scanning range of *m/z* = 200–2000. An MS/MS experiment (MS2) was conducted with a resolution of 17,500, a separation window of *m/z* = 3.0 (N) CE of 13 V, and a default charge state of 1.

### 2.8 Nuclear Magnetic Resonance Spectroscopy Method

First, the cefotaxime sodium, its dimer and trimer were separately dissolved in DMSO-*d*6 to a concentration of approximately 20, 20 and 15 mg/ml respectively, and tetramethylsilane (TMS) was added as the internal standard peak (δ_H_ 0.00 and δ_C_ 0.00). The operating frequencies of the ^1^H and ^13^C NMR were 600.13 and 150.90 MHz, respectively, the experimental temperature was 24.5°C, and the spectral widths were 11,904.76 and 35,714.29 Hz, respectively. The operating frequency of the attached proton test (APT) spectrum was 150.90 MHz, and the spectral width was 35,714.29 Hz. Bruker standard pulse sequence was used for the ^1^H–^1^H correlated spectroscopy (COSY), ^1^H-^13^C heteronuclear single quantum (HSQC), and ^1^H-^13^C heteronuclear multiple bond correlation (HMBC) experiments. The spectral widths of F2 (^1^H) and F1 (^1^H) of ^1^H–^1^H COSY were both 7,812.50 Hz, the sampling data array was t2 × t1 = 2048 × 128, and the number of transients averaged was 4. The F2 (^1^H) and F1 (^13^C) spectral widths of HSQC and HMBC were 7,812.50 and 33,201.94 Hz, respectively, the sampling data array was t2 × t1 = 4,096 × 128, and the number of transients averaged was 8. The NMR spectra were processed using the Mestrenova 14.0.0 software.

### 2.9 Polymer Stock Solution Preparation

A concentrated solution degradation method:Approximately 2.0 g of cefotaxime raw material was transferred into a 100-ml volumetric flask, and distilled water was added to dissolve and dilute it to a concentration of approximately 20 mg/ml. The mixture was shaken well to produce a solution, after which it was allowed to stand at 25 ± 2°C for 7 days. It was then freeze-dried and stored for future use.

## 3 Results and Discussion

### 3.1 Analysis of Polymer Stock Solution by RP-HPLC

**FIGURE 2:**
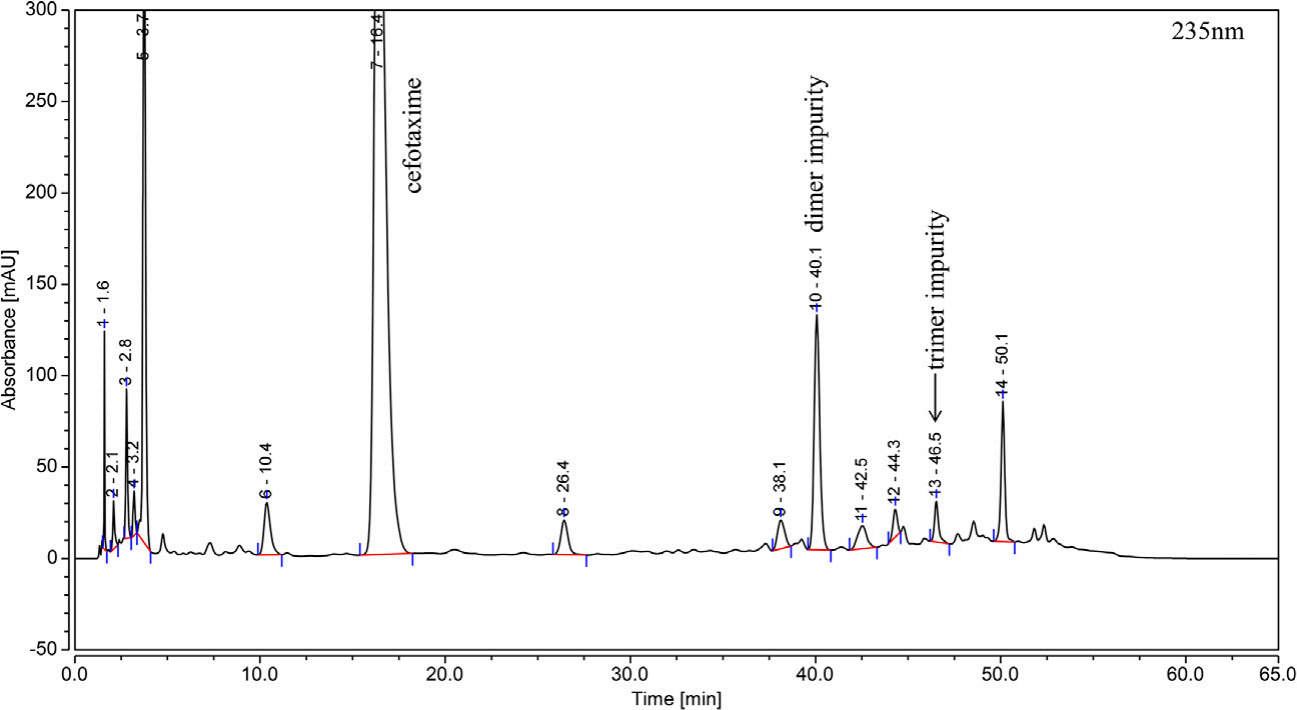
Typical RP-HPLC chromatogram of cefotaxime polymer stock solution.

By applying the method described in Section 2.3, the polymer stock solution was analyzed, and the dimer and trimer impurities were detected in the chromatogram, as shown in Figure 2.

### 3.2 UV Spectrum Analysis

The UV spectra in Supplementary Figure S1B show that the maximum absorption of the cefotaxime dimer occurred at 237 and 257 nm, and the minimum absorption occurred at 218 nm. Based on the position, intensity, and molar absorption coefficient of the absorption peak, it was preliminarily inferred that the absorption peak at 257 nm (ε: 318,462) was caused by the π-π* transition of the double bond on the six-ring matrix. The absorption peak (ε: 329,231) at 237 nm was caused by the K-band absorption of the aminotaxime conjugation system. Compared with cefotaxime sodium (Supplementary Figure S1A), the absorption peak of the conjugated K-band in the dimer shifted from 235 to 237 nm, indicating that the K-band conjugation system in the dimer was longer and the amino group at position 17 may have undergone a substitution reaction.

Similar to the cefotaxime dimer, the cefotaxime trimer (Supplementary Figure S1C) exhibited maximum absorption at 237 and 255 nm, and the minimum absorption was at 218 nm. It was preliminarily inferred that the absorption peak at 255 nm (ε: 354,167) was caused by the π-π* transition of the double bond on the six-ring matrix. The absorption peak at 237 nm (ε: 373,167) was the K-band absorption of the aminotaxime conjugated system. Compared with cefotaxime sodium, the cefotaxime trimer also showed a red shift in the absorption peak of the conjugated K band at 237 nm, and it was speculated that the amino groups of both molecules of aminotaxime in the cefotaxime trimer were substituted.

### 3.3 IR Spectrum Analysis

The IR spectrum in Supplementary Figure S2B shows that the dimer had 10 characteristic IR absorption peaks. The peak at 1768 cm^−1^ was assigned to carbonyl stretching vibrations of the β-lactam ring. The peak at 1728 cm^−1^ was assigned to the carbonyl stretching vibrations of the acetyl group. The peak at 1,669 cm^−1^ was assigned to C=N stretching vibrations of C=N-OCH_3_. The peak at 1,624 cm^−1^ was assigned to C=O stretching vibrations of COOH. The peak at 1,531 cm^−1^ was assigned to N-H bending vibrations and C-N stretching vibrations. The peaks at 1,384 and 1,351 cm^−1^ were assigned to CH_3_ stretching vibrations. The peaks at 1,232 and 1,037 cm^−1^ were assigned to the stretching vibrations of CO-O-R and C-O on the three side chains, respectively. The peak at 1,110 cm^−1^ was assigned to C-N stretching vibrations.

The dimer (Supplementary Figure S2C) had nine characteristic IR absorption peaks. The peak at 1766 cm^−1^ was assigned to the carbonyl stretching vibrations of the β-lactam ring. The peak at 1727 cm^−1^ was assigned to the carbonyl stretching vibrations of the acetyl group. The peak at 1,670 cm^−1^ was assigned to the C=N stretching vibrations of C=N-OCH_3_. The peak at 1,626 cm^−1^ was assigned to the C=O stretching vibrations of COOH. The peak at 1,531 cm^−1^ was assigned to the amide N-H bending vibrations and C-N stretching vibrations. The peak at 1,362 cm^−1^ was assigned to the bending vibrations of CH_3_. The two spectral bands at 1,232 and 1,036 cm^−1^ were assigned to the stretching vibrations of CO-O-R and C-O on the three side chains, respectively, and the peak at 1,109 cm^−1^ was assigned to C-N stretching vibrations.

The above IR spectrum analysis showed that the IR spectra of the cefotaxime dimer and trimer were basically the same as that of cefotaxime sodium (Supplementary Figure S2A), indicating that they contained similar functional groups (Table 1).

**TABLE 1:** IR spectra of cefotaxime Dimer and cefotaxime Trimer.

Wavenumbers (cm^−1^)		Mode of vibration	Attribution of absorption peak
Cefotaxime dimer	Cefotaxime trimer		
1768	1766	V_C=O_	C=O of lactam
1728	1727	V_C=O_	CH_3_C-(O)-O
1,669	1,670	V_C=N_	C=N-OCH_3_
1,624	1,626	V_C=O_	C-(O)-OH
1,531	1,531	δ_NH_ V_C-N_	O=C-NH
1,384/1,351	1,362	δ_CH_	-CH_3_
1,232	1,232	V_CO_	-CO-O-R
1,107	1,109	V_CN_	-C-N
1,037	1,036	V_CO_	-C-O

### 3.4 High-Resolution Mass Spectroscopy Analysis

**FIGURE 3:**
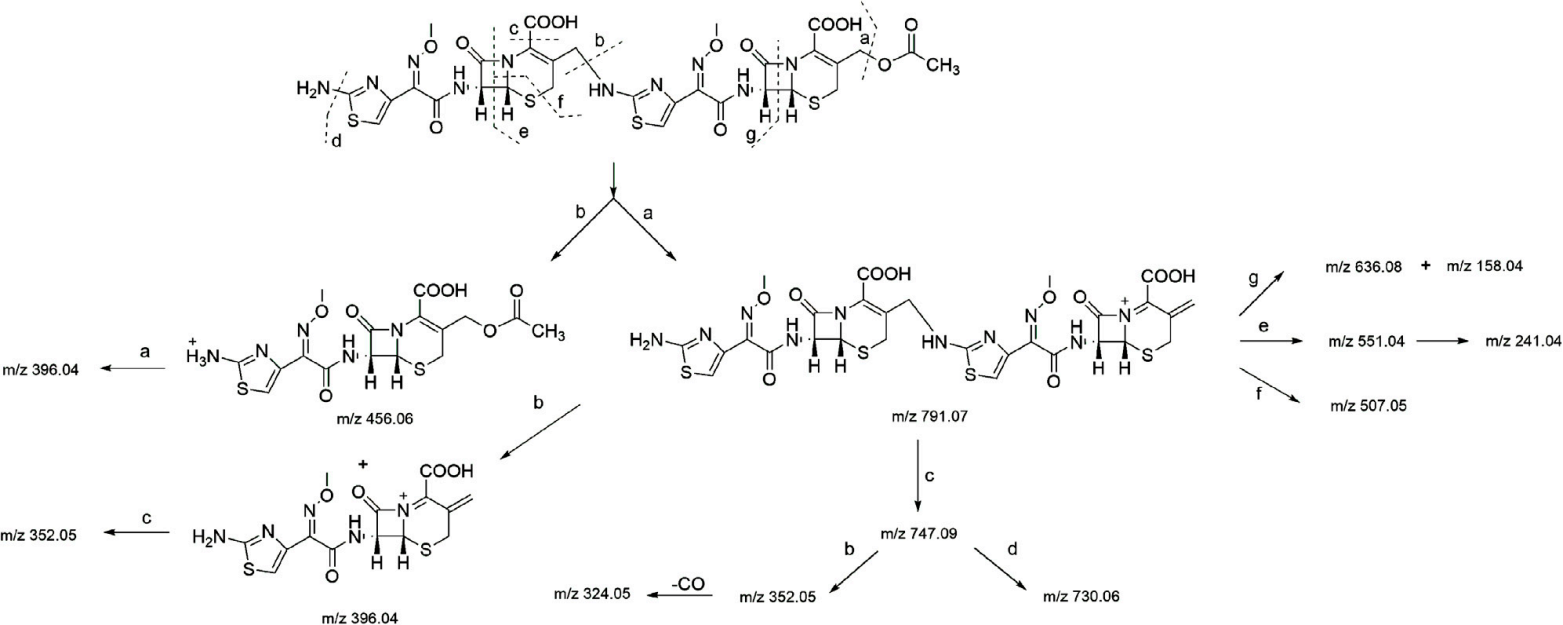
MS fragmentation pathway of cefotaxime dimer.

In the HR-MS spectra of the dimer (Supplementary Figure S3A), the adduct ion peaks of *m/z* = 851.088 87 [M + H]^+^ and *m/z* = 426.048 28 [M+2H]^2+^ were detected under positive mode, which indicated that the relative molecular weight of the dimer was 850.09192 amu, and the annotated molecular formula was C_30_H_30_N_10_O_12_S_4._ The relative error between the experimental and theoretical molecular weight values was −0.8300 ppm. The results showed that the dimer had one C_2_H_4_O_2_ group fewer than two molecules of cefotaxime (molecular formula: C_16_H_17_N_5_O_7_S_2_, relative molecular weight: 455.05694 amu), which was determined to be the side-chain substituent (acetoxy) of cefotaxime. The secondary mass spectrum (Supplementary Figure S3B) showed that the main cleavage ions of the [M + H]^+^ peak included *m/z =* 791.07, 747.09, 730.06, 636.08, 551.04, 507.08, 456.06, 396.04, 352.05, and 241.04. The specific cleavage pathway is shown in Figure 3. It was preliminarily assumed that the amino group at position seven of one molecule of cefotaxime formed a σ bond with the carbon atom a t the 3α position of another molecule of cefotaxime, and a polymerization reaction occurred to form the cefotaxime dimer.

**FIGURE 4:**
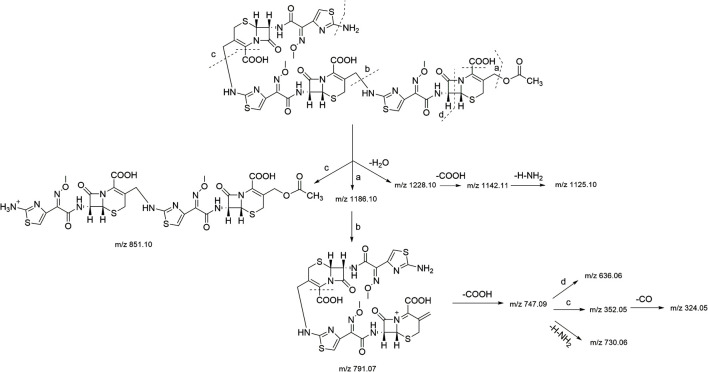
MS fragmentation pathway of cefotaxime trimer.

In the HR-MS spectra of the trimer (Supplementary Figure S4A), adduct ion peaks with *m/z* = 1,246.13464 [M + H]^+^, 623.57159 [M + H]^2+^, and 416.05014 [M+2H]^3+^ were detected, which indicated that the relative molecular weight of the trimer was 1,245.12891 amu, and the annotated molecular formula was C_44_H_43_N_15_O_17_S_6_. The relative error between the experimental and theoretical molecular weight values was −0.3500 ppm. Similar to the dimer, the trimer had two C_2_H_4_O_2_ groups fewer than three molecules of cefotaxime, both of which were determined to be the side-chain substituents (acetoxy) of cefotaxime. The secondary mass spectrum (Supplementary Figure S4B) showed that the main cleavage ions of the [M + H]^+^ peak included *m/z =* 1,228.10, 1,186.10, 1,142.11, 1,125.10, 851.10, 791.07, 747.09, 730.06, 636.06, 352.05, and 324.05. The specific fragmentation pathway assignment is shown in Figure 4. It was preliminarily assumed that the amino group at position seven of one molecule of cefotaxime formed a σ bond with the carbon atom at position 3α of one molecule of the cefotaxime dimer, and it polymerized to generate the cefotaxime trimer. In the meanwhile, we could conclude from the above results that there was essentially no significant difference in fragmentation pathways in the secondary mass spectra of dimers and trimers.

### 3.5 NMR Analysis

**FIGURE 5:**
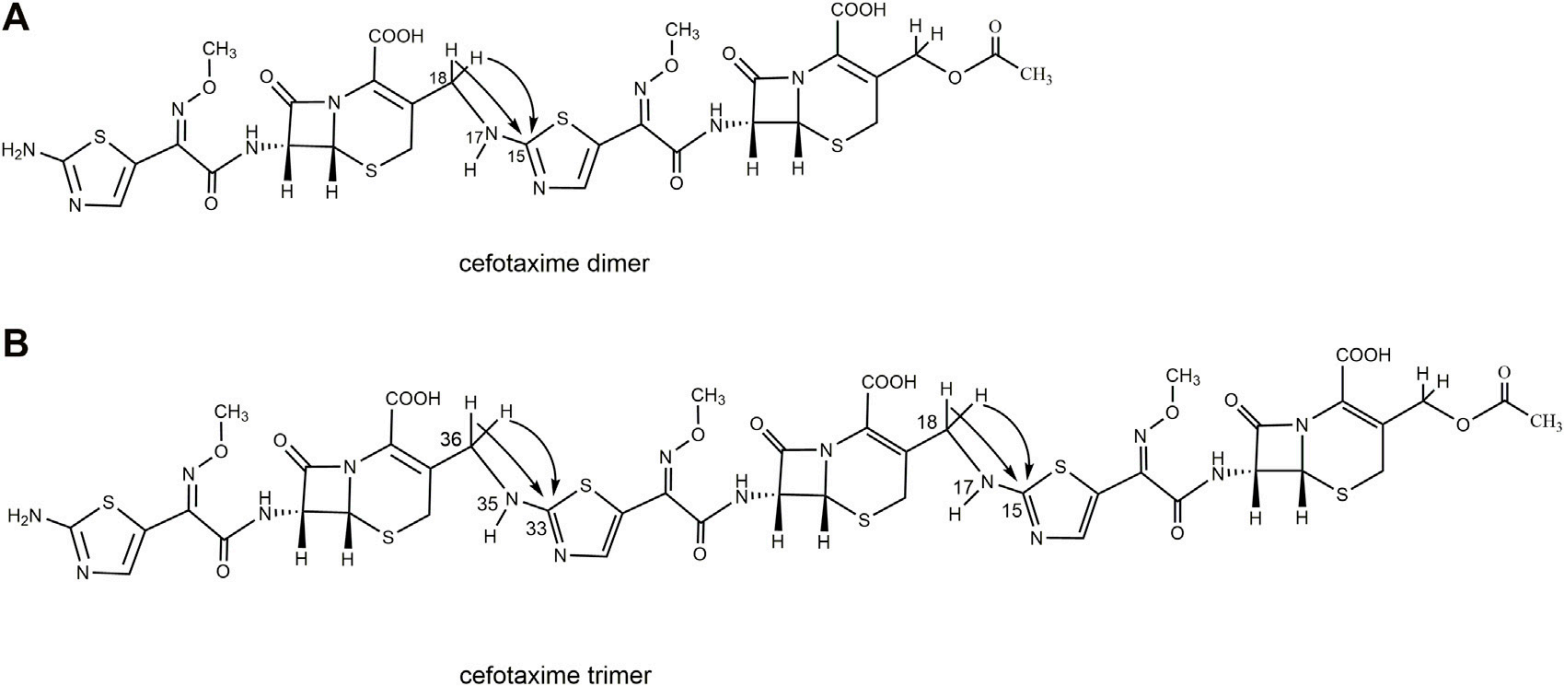
The scheme of partial HMBC correlation ship of cefotaxime dimer and trimer.

**FIGURE 6:**
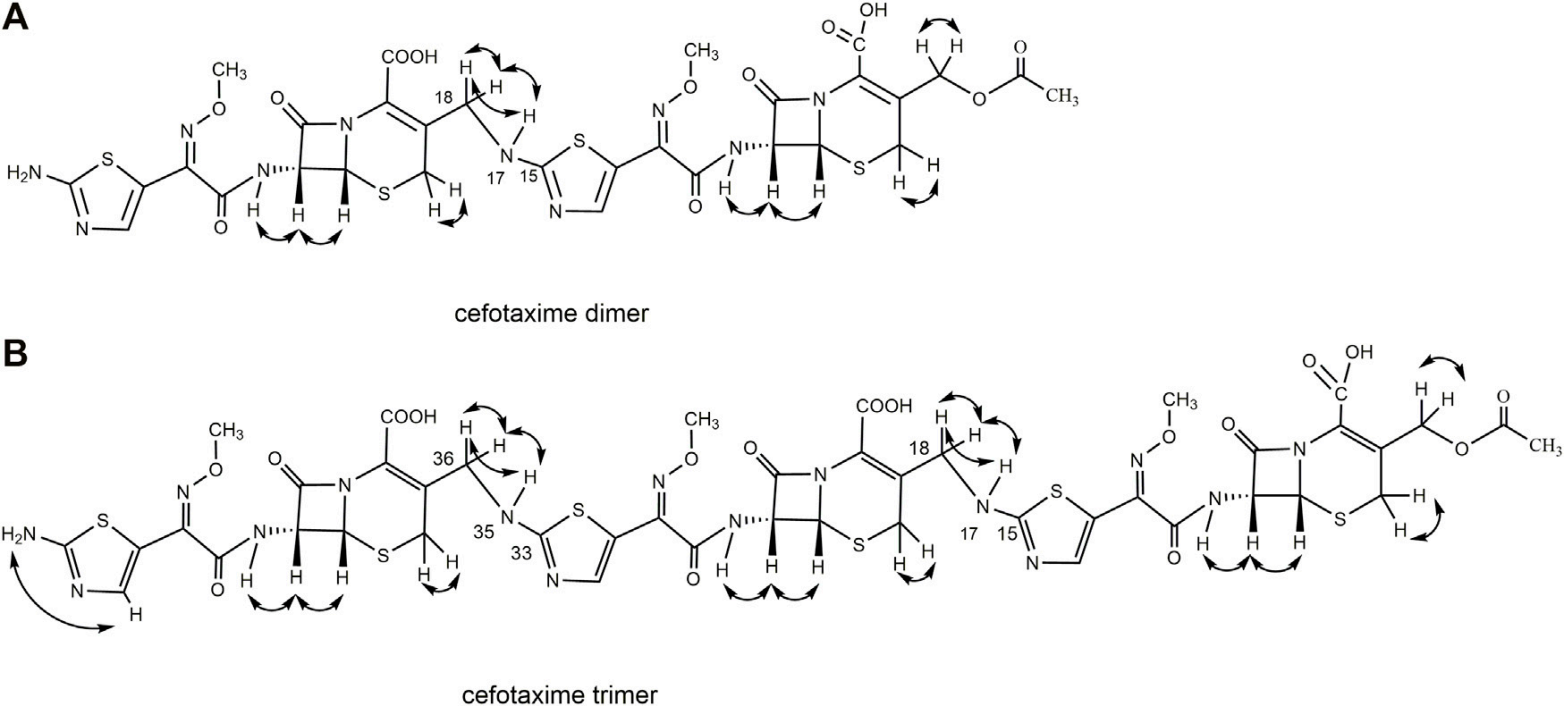
The scheme of ^1^H–^1^H COSY correlation ship of cefotaxime dimer and trimer.

Based on the one-dimensional (1D) and two-dimensional (2D) NMR spectra (Supplementary Figures S5–S16), the proton and carbon signals of cefotaxime sodium and its dimer and trimer impurities were assigned, and the connection sites and the absolute configurations of the cefotaxime dimer and trimer were identified. Based on the analysis of the data from the ^1^H NMR and ^13^C NMR spectra of cefotaxime sodium, its dimer and trimer impurities are presented in Tables 2–5, and the corresponding HMBC and ^1^H–^1^H COSY assignments are shown in Figures 5, 6, respectively.

**TABLE 2:** ^1^H-NMR and 2D-NMR spectra of cefotaxime and cefotaxime Dimer.

^1^H No	Cefotaxime dimer	HMBC	^1^H–^1^H COSY	Cefotaxime sodium	Cefotaxime sodium
	(Experiment value)	(Experiment value)	(Experiment value)	(References value) Li et al. (2021d)	(Experiment value)
3α-a	4.69(1H,d,J = 12.8 Hz)	C1’, C2, C3, C4	H3α-b	4.76(1H,d,J = 12.8 Hz)	4.77(1H,d,J = 12.0 Hz)
3α-b	4.99(1H,d,J = 12.8 Hz)	C1’, C2, C3, C4	H3α-a	4.92(1H,d,J = 12.4 Hz)	4.99(1H,d,J = 12.0 Hz)
4-a	3.48(1H,d,J = 18.2 Hz)	C3α, C6, C2, C3	H4-b	3.71(1H,d,J = 18.0 Hz)	3.47(1H,d,J = 17.3 Hz)
4-b	3.62(1H,d,J = 18.2 Hz)	C6, C2, C3	H4-a	3.44(1H,d,J = 18.0 Hz)	3.21(1H,d,J = 17.3 Hz)
6	5.15(1H,d,J = 4.8 Hz)	C8	H7	5.20(1H,d,J = 4.8 Hz)	5.01(1H,d,J = 4.8 Hz)
7	5.76(1H,dd,J_1_ = 4.8 Hz, J_2_ = 8.2Hz)	C6,C8	H6, 9-NH	5.85(1H,d,J = 4.4 Hz)	5.59(1H,dd,J_1_ = 4.8 Hz, J_2_ = 8.2Hz)
9-NH	9.62(1H,d,J = 8.1 Hz)^a^	C7,C10	H7	—	9.52(1H, d, J = 8.2 Hz)^a^
11-NOCH_3_	3.86 (3H. s.)	\	\	4.03(3H,s)	3.83(3H,s)
13	6.83 (1H, s.)	C11,C12,C15	\	7.04 (1H,s.)	6.73 (1H,s.)
17-NH	8.12 (1H,m\)^a^	\	H18-a,H18-b	—	7.25(2H,s.)^a^
2-COOH	13.66 (1H, br.)^b^	\	\	—	—
2′-CH_3_	2.03(3H. s.)	C1′	\	—	2.00(3H.s.)
18-a	4.19(1H,dd,J_1_ = 15 Hz,J_2_ = 5.0 Hz)	C19,C20,C24,C15	H18-b, 17-NH	—	—
18-b	4.30(1H,dd,J_1_ = 15 Hz,J_2_ = 6.2 Hz)	C19,C20,C24,C15	H18-a,17-NH	—	—
20-a	3.51(1H,d,J = 18.2 Hz)	C18,C19,C22,C24	H20-b	—	—
20-b	3.62(1H,d,J = 18.2 Hz)	C19,C22,C24	H20-a	—	—
22	5.11(1H,d,J = 4.8 Hz)	C26	H25	—	—
25	5.79(1H,dd,J_1_ = 4.8 Hz, J_2_ = 8.2Hz)	C22,C26	H22,27-NH	—	—
27-NH	9.59(1H, d, J = 8.1 Hz)^a^	C25,C28	H25	—	—
29-NOCH_3_	3.83 (3H. s.)	\	\	—	—
31	6.73(1H, s.)	C29, C30, C33	\	—	—
35-NH_2_	7.21(2H, s.)^a^	\	\	—	—
24-COOH	13.66 (1H, br.)^b^	\	\	—	—

—;, not available; \: not detected at all.

a Partial detected after D_2_O exchange.

b Not detected after D_2_O exchange.

**TABLE 3:** ^13^C-NMR and APT spectra of cefotaxime and cefotaxime Dimer.

^13^C No	Cefotaxime dimer	Carbon type	Cefotaxime sodium	Cefotaxime sodium
	(Experiment value)		(References value) Li et al. (2021d)	(Experiment value)
2	126.9	C	131.7	135.7
2-COOH	163.5	C	168.6	164.5
3	123.9	C	113.5	112.1
3α	63.2	CH_2_	64.3	65.0
4	26.2	CH_2_	25.8	25.8
6	57.9	CH	57.3	57.8
7	59.1	CH	59.0	58.5
8	164.2	C	164.0	162.6
10	163.3	C	165.0	163.5
11	149.3	C	148.0	149.6
11-NOCH_3_	62.5	CH_3_	62.9	62.3
12	142.8	C	140.3	143.1
13	109.8	CH	116.5	109.4
15	169.0	C	171.1	168.9
18	45.5	CH_2_	—	—
19	125.9	C	—	—
20	26.9	CH_2_	—	—
22	58.0	CH	—	—
24	127.9	C	—	—
24-COOH	163.5	C	—	—
25	59.1	CH	—	—
26	164.2	C	—	—
28	163.4	C	—	—
29	149.5	C	—	—
29-NOCH_3_	62.4	CH_3_	—	—
30	143.0	C	—	—
31	109.4	CH	—	—
33	168.9	C	—	—
1′	170.7	C	—	171.0
2′-CH_3_	21.1	CH_3_	—	21.2

—, not available.

**TABLE 4:** ^1^H-NMR and 2D-NMR spectra of cefotaxime and cefotaxime Trimer.

^1^H No	Cefotaxime trimer	HMBC	^1^H–^1^H COSY	Cefotaxime sodium	Cefotaxime dimer
	(Experiment value)	(Experiment value)	(Experiment value)	(Experiment value)	(Experiment value)
3α-a	4.69(1H,d,J = 12.8Hz)	C1’, C2, C3, C4	H3α-b	4.77(1H,d,J = 12.0Hz)	4.69(1H,d,J = 12.8Hz)
3α-b	4.99(1H,d,J = 12.8Hz)	C1’, C2, C3, C4	H3α-a	4.99(1H,d,J = 12.0Hz)	4.99(1H,d,J = 12.8Hz)
4-a	3.47(1H,d,J = 18Hz)	C3α, C6, C2, C3	H4-b	3.47(1H,d,J = 17.3Hz)	3.48(1H,d,J = 18.2Hz)
4-b	3.62(1H,d,J = 18Hz)	C2, C3	H4-a	3.21(1H,d,J = 17.3Hz)	3.62(1H,d,J = 18.2Hz)
6	5.15(1H,d,J = 4.8Hz)	C8	H7	5.01(1H,d,J = 4.8Hz)	5.15(1H,d,J = 4.8Hz)
7	5.77(1H,m.)	C8	H6, 9-NH	5.59(1H,dd,J_1_ = 4.8Hz, J_2_ = 8.2Hz)	5.76(1H,dd,J_1_ = 4.8Hz, J_2_ = 8.2Hz)
9-NH	9.59(1H,d,J = 8.0Hz)^a^	C7,C10	H7	9.52(1H, d, J = 8.2Hz)^a^	9.62(1H,d,J = 8.1Hz)^a^
11-NOCH_3_	3.86 (3H. s.)	\	\	3.83(3H,s)	3.86 (3H. s.)
13	6.83 (1H, s.)	C11,C12,C15	\	6.73(1H,s.)	6.83 (1H, s.)
17-NH	8.12(1H, br.)^a^	\	H18-a,H18-b	7.25(2H,s.)^a^	8.12(1H,m.)^a^
2-COOH	13.64 (1H, br.)^b^	\	\	−	13.66 (1H, br.)^b^
2′-CH_3_	2.03(3H. s.)	C1′	\	2.00(3H.s.)	2.03(3H. s.)
18-a	4.18(1H,m.)	\	H18-b, 17-NH	—	4.19(1H,dd,J_1_ = 15Hz,J_2_ = 5.0Hz)
18-b	4.30(1H,m)	C19,C20,C24,C15	H18-a,17-NH	—	4.30(1H,dd,J_1_ = 15Hz,J_2_ = 6.2Hz)
20-a	3.51(1H,d,J = 18Hz)	C18,C19,C22,C24	H20-b	—	3.51(1H,d,J = 18.2Hz)
20-b	3.47(1H,d,J = 18Hz)	C19,C24	H20-a	—	3.62(1H,d,J = 18.2Hz)
22	5.11(1H,m.)	C26	H25	—	5.11(1H,d,J = 4.8Hz)
25	5.77(1H,m.)	C26	H22,27-NH	—	5.79(1H,dd,J_1_ = 4.8Hz, J_2_ = 8.2Hz)
27-NH	9.62(1H,d,J = 8.0Hz)^a^	C25,C28	H25	—	9.59(1H, d, J = 8.1Hz)^a^
29-NOCH_3_	3.85 (3H. s.)	\	\	—	3.83 (3H. s.)
31	6.82(1H, s.)	C29, C30, C33	\	—	6.73(1H, s.)
35-NH	8.12(1H,br.)^a^	\	H36-a,H36-b	—	7.21(2H, s.)^a^
24-COOH	13.64 (1H, br.)^b^	\	\	—	13.66 (1H, br.)^b^
36-a	4.18(1H,m)	\	H36-b, 35-NH	—	—
36-b	4.30(1H,m)	C33,C38,C37,C42	H36-a,35-NH	—	—
38-a	3.51(1H,d,J = 18Hz)	C36,C38,C40,C42	H38-b	—	—
38-b	3.63(1H,d,J = 18Hz)	C38, C42	H38-a	—	—
40	5.11(1H,m.)	C44	H43	—	—
43	5.77(1H,m.)	C44	H40, 45-NH	—	—
45-NH	—	C43,C46	H43	—	—
47-NOCH3	3.83 (3H. s.)	\	\	—	—
49	6.73(1H, s.)	C47,C48,C51	53-NH_2_	—	—
53-NH2	7.20 (2H. s.)^a^	\	H49	—	—
42-COOH	—	\	\	—	—

—, not available; \: not detected at all.

a Partial detected after D_2_O exchange.

b Not detected after D_2_O exchange.

**TABLE 5:** ^13^C-NMR and APT spectra of cefotaxime and cefotaxime Trimer.

^13^C No	Cefotaxime trimer	Carbon type	^13^C No	Cefotaxime trimer	Carbon type
	(Experiment value)			(Experiment value)	
2	127.1	C	28	163.4	C
2-COOH	163.7	C	29	149.5	C
3	123.7	C	29-NOCH_3_	62.5	CH_3_
3α	63.2	CH_2_	30	142.8	C
4	26.2	CH_2_	31	109.9	CH
6	58.0	CH_2_	33	169.0	C
7	59.1	CH_2_	36	45.6	CH_2_
8	164.2	C	37	126.0	C
10	163.4	C	38	26.9	CH_2_
11	149.3	C	40	57.9	CH
11-NOCH_3_	62.5	CH_3_	42	127.7	C
12	142.8	C	42-COOH	163.5	C
13	109.7	CH	43	59.1	CH
15	169.0	C	44	164.1	C
18	45.6	CH_2_	46	163.4	C
19	126.0	C	47	149.5	C
20	26.9	CH_2_	47-NOCH_3_	62.4	CH_3_
22	57.9	CH	48	143.0	C
24	127.7	C	49	109.4	CH
24-COOH	163.5	C	51	168.9	C
25	59.1	CH	1′	170.7	C
26	164.1	C	2′-CH_3_	21.1	CH_3_

—, not available

A total of 30 proton signals were detected in the ^1^H NMR spectrum of the dimer impurity and 30 carbon signals in the ^13^C NMR spectrum of the dimer. According to analysis, the 17-NH_2_ of cefotaxime sodium was a primary amino group, while the 17-NH of the dimer was a secondary amino group, indicating that one amino proton of the latter was replaced. In the ^1^H-NMR spectrum, the 17-NH proton of the dimer was not a single peak but multiple peaks, which indicated that this proton was coupled with other protons. In the ^1^H–^1^H COSY spectrum, the 17-NH proton (δ8.12, m) of the dimer had a correlation peak with H18 (2H, δ4.19 and δ4.30). In the HMBC spectrum, the two protons of H18 [δ4.19 (1H, dd, *J*_1_ = 15 Hz, *J*_2_ = 5.0 Hz) and δ4.30 (1H, dd, *J*_1_ = 15 Hz, *J*_2_ = 6.2 Hz)] were remotely correlated with C15 (δ169.0). These results indicated that the dimer was connected by σ bonds between the 17-NH and 18-CH_2_ groups.

A total of 41 proton signals were detected in the ^1^H NMR spectrum of the trimer impurity. The^13^C NMR spectrum of the trimer should show 44 carbon signals, but only 31 carbon signals were actually detected, possibly because the chemical environment contribution to the C36–C51 carbon signals in the trimer was similar to the chemical environment contribution to the C3–C33 carbon signals. This resulted in partial overlap of the carbon signals in the carbon spectrum. This overlap included three groups of CH_3_ carbon signals at δ21.1, δ62.5, and δ62.4, which were assigned to 2′-CH_3_, 11-OCH_3_ (29-OCH3), and 47-OCH_3_, respectively; four groups of CH_2_ carbon signals at δ26.2, δ63.2, δ26.9, and δ45.6, which were assigned to C4, C3α, C20 (C38), and C18 (C36), respectively; and seven groups of CH carbon signals at δ58.0, δ57.9, δ59.1, δ59.1, δ109.7, δ109.9, and δ109.4, which were assigned to C6, C22 (C40), C7, C25 (C43), C13, C31, and C49, respectively, according to the HSQC spectrum and the chemical shift rule of the carbon spectrum. The remaining groups of C signals were assigned according to the HMBC spectrum and the carbon spectrum data of the dimer, as shown in Table 5. Thus, all 41 proton signals and 44 carbon signals were assigned. According to analysis, the 17-NH_2_ of cefotaxime sodium was a primary amino group, while the 17-NH in the dimer and the 35-NH in the trimer were secondary amino groups, indicating that two amino protons of the trimer were replaced. In the ^1^H–^1^H COSY spectrum, the 17-NH (δ8.12, m) and 35-NH (δ8.12, m) protons of the trimer had correlation peaks with H18 (2H, δ4.18 and δ4.30) and H36 (2H, δ4.18 and δ4.30). In the HMBC spectrum, the two protons of H18 [δ4.30 (1H, m)] and H36 [δ4.30 (1H, m)] were remotely correlated with C15 (δ169.0) and C33 (δ169.0). Furthermore, the molecular formulas of unsaturated cefotaxime sodium, its dimer, and its trimer were Ω = 11, 21, and 31, respectively. These results indicated that the trimer was connected by the σ bonds between the 17-NH and 18-CH_2_ groups and between the 35-NH and 36-CH_2_ groups.

In theory, there were four double-bonded cis/trans isomers in the dimer (11Z vs. 29Z, 11Z vs. 29E, 11E vs. 29E, and 11E vs. 29E) and eight double-bonded cis/trans isomers in the trimer (11Z vs. 29Z vs. 47Z, 11Z vs. 29Z vs. 47E, 11Z vs. 29E vs. 47E, 11Z vs. 29E vs. 47Z, 11E vs. 29Z vs. 47Z, 11E vs. 29Z vs. 47E, 11E vs. 29E vs. 47Z, and 11E vs. 29E vs. 47E). In a previous study, it was found that when the double bond configuration of amino thiaoxime changed from Z to E, the chemical shift of the protons on the five-membered thiazole ring shifted from high field to low field by about 0.5–0.7 ppm, and the carbon chemical shift of the amino thiaoxime methoxyl group shifted from high field to low field by 0.3–0.9 ppm (Li et al., 2021d). It is known that the double bond configuration at position 11 of cefotaxime sodium is Z, the chemical shift of H13 is δ6.73, and the carbon chemical shift of 11-OCH3 is δ62.3. The chemical shifts of protons H13 and H31 in the dimer were δ6.83 and δ6.73, respectively, which were basically consistent with the chemical shift of H13 in cefotaxime sodium. There was no evident shift from the high field to the low field. The carbon chemical shifts of 11-OCH_3_ and 29- OCH_3_ in the dimer were δ62.5 and δ62.4, respectively. Compared with cefotaxime sodium, the dimer slightly shifted to the low field only by 0.1–0.2 ppm. The above analysis showed that the configurations of the two double bonds at positions 11 and 29 in the dimer were all Z-types. The chemical shifts of protons H13, H31, and H49 in the trimer were δ6.83, δ6.83, and δ6.73, respectively, which were basically consistent with the chemical shifts of H13 and H31 in the dimer, and there was no evident shift from the high field to the low field. The carbon chemical shifts of 11-OCH_3_, 29-OCH_3_, and 47- OCH_3_ in the trimer were δ62.5, δ62.5, and δ62.4, respectively. Compared with the dimer, there was also no evident shift. Hence, the configurations of the three double bonds at positions 11, 29, and 47 in the trimer were all Z-types.

There were four chiral centers in the dimer, including C6, C7, C22, and C25. The four carbon atoms were all tertiary carbons; so, we could infer whether their absolute configurations changed by the proton coupling constant. The data in Table 2 show that the coupling constants of H6 vs. H7 and H22 vs. H25 in the dimer were all 4.8 Hz, which were the same as that of H6 vs. H7 in cefotaxime sodium. Hence, the two β-lactam rings were stable, the absolute configurations of C6 and C22 in the dimer were the same as that of C6 in cefotaxime sodium, and the absolute configurations of C7 and C25 in the dimer were the same as that of C7 in cefotaxime sodium. The absolute configurations of the four chiral centers were all R-types. There were six chiral centers in the trimer: C6, C7, C22, C25, C40, and C43. The data in Table 3 show that the coupling constant of H6 vs. H7 were *J* = 4.8 Hz, those of H22 vs. H25 were *J* = 4.7 and 4.8 Hz, respectively, and those of H40 vs. H43 were *J* = 4.7 and 4.8 Hz, respectively, all of which were similar to those of H6 vs. H7 and H22 vs. H25 in the dimer. These results indicated that the three β-lactam rings were stable; so, the absolute configurations of cefotaxime trimers C6, C22, and C40 in the trimer were the same as those of C6 and C22 in the dimer. Furthermore, the absolute configurations of C7, C25, and C43 were the same as those of C7 and C25 in the dimer. The absolute configurations of the six chiral centers were all R types.

Therefore, the dimer impurity from the actual sample of industrial medicines was named (6R, 7R)-3-(acetoxymethyl)-7-[(Z)-2-(2-(6R, 7R))-7-(Z)-2-((2-aminothiazol-4-yl)-2-(methoxyimino) acetamido)-2-carboxy-8-oxo-5-thia-1-azabicyclo [4.2.0]oct-2-en-3-yl)methyl)amino)thiazol-4-yl)-2-(methoxyimino)acetamido]-8-oxo-5-thia-1-azabicyclo [4.2.0] oct-2-ene-2-carboxylic acid, which is considered the same as the dimer investigated (Agarwal et al., 2004) and published in PubChem. And the trimer impurity from the actual sample of industrial medicines was named (6R, 7R)-3-(acetoxymethyl)-7-[(Z)-2-(2-(6R, 7R)-7-(Z)-2-(2-(6R, 7R)-7-((Z)-2-(2-aminothiazol-5-yl)-2-(methoxyimino) acetamido]-2-carboxy-8-oxo-5-thia-1-azabicyclo [4.2.0]oct-2-en-3-yl)methyl)amino)thiazol-5-yl)-2-(methoxyimino)acetamido)-2-carboxy-8-oxo-5-thia-1-azabicyclo [4.2.0]oct-2-en-3-yl)methyl)amino)thiazol-5-yl)-2-(methoxyimino)acetamido]-8-oxo-5-thia-1-azabicyclo [4.2.0]oct-2-ene-2-carboxylic acid.

## 4 Conclusion

HPSEC preparation and RP-HPLC were used to prepare a concentrated degraded polymer solution for two-step purification and separation in this study. Combined with the characteristics of short preparation times of the HPSEC preparation method and the high separation effect of the RP-HPLC preparation method, the efficiency of separation and purification could be significantly improved. The UV, IR, HR-MS, 1D NMR, and 2D NMR spectra of cefotaxime sodium, its dimer, and its trimer were determined. The characteristic absorption peaks in the UV and IR spectra were assigned. The molecular weights and molecular formulas of the dimer and trimer impurities were determined by HR-MS, and the characteristic structural fragments were deduced by secondary mass spectrometry. All the ^1^H-NMR and ^13^C-NMR signals were assigned by 2D NMR techniques. The above data confirmed the chemical structure of the dimer from the actual sample of industrial medicines is the same as in the dimers investigated earlier. And the connection sites and the absolute configurations of trimer impurity for the first time. These results have great significance for the study of the polymerization sites and quality control of the polymer impurities in β-lactam antibiotics.

## Data Availability

The original contributions presented in the study are included in the article/Supplementary Material, further inquiries can be directed to the corresponding author.

## References

[B1] Agarwal S. Bhatnagar U. Rajesh N. (2004). Acute and Genotoxic Profile of a Dimeric Impurity of Cefotaxime. [J]. Int. J. Toxicol. 23, 41–45. 10.1080/10915810490265441 15162846

[B2] Anacona J. R. Osorio I. (2008). Synthesis and Antibacterial Activity of Copper(II) Complexes with Sulphathiazole and Cephalosporin Ligands. Transit. Met. Chem. 33, 517–521. 10.1007/s11243-008-9074-y

[B3] Buonomo A. Pascolini L. Rizzi A. (2016). Cross-reactivity and Tolerability of Ertapenem in Patients with Ig E-Mediated Hypersensitivity to β-Lactams. J. Investig. Allergol. Clin. Immunol. 26, 100–105. 10.18176/jiaci.0019 27164625

[B4] Chinese Pharmacopoeia Commission (2020). Pharmacopoeia of the People's Republic of China ( 2020 Edition Volume II). Beijing: Chemical Industry Press, 362.

[B5] Chong X.-M. Tian Y. Yao S.-C. (2021). Comparison of Determination Methods for Cefathiamidine Polymer for Injection and Analysis of Polymer Impurities. Chin. J. New Drug 30, 1334–1339. 10.3969/j.issn.1003-3734.2021.14.013

[B6] Department of Medical Administration. (2021) .Notice of the General Office of the National Health and Family Planning Commission on Printing and Issuing the Guidelines for Skin Testing of β-Lactam Antibacterial Drugs. Available at: http://www.nhc.gov.cn/yzygj/s2021/202104/a33f49b8c4b5421c85a5649a28a0fce2.shtml . (Accessed April 16, 2021).

[B7] Dong J. Li S.-H. Wang Y.-Q. (2015). Determination of Polymer in Cefotaxime Sodium and Tazobactam Sodium for Injection (6:1) by HPSEC [J]. Chin. Pharm. 27, 3841–3843. 10.6039/j.issn.1001-0408.2015.27.35

[B8] European Directorate for the Quality of Medicine & Health Care (2020). The European Pharmacopoeia 10.0. Strasbourg: European Directorate for the Quality of Medicines & Health Care, 2122–2123.

[B9] Hu C.-Q. (2020). Formation and Development of Impurity Control Strategy for β-Lactam Antibiotic Polymers. Chin. J. New Drug 29, 1231–1244. 10.3969/j.issn.1003-3734.2020.11.007

[B10] Hu C.-Q. (2008). Prospect of β-lactam Antibiotic Polymer Analysis Technology. Chin. J. New Drug 17, 2098–2102. 10.3321/j.issn:1003-3734.2008.24.006

[B11] Hu W.-G. Han B. Hu L.-M. (2020). Identification of High Molecular Impurities in Cefathiamidine for Injection by 2D-LC-IT-TOF/MS Combined with Cef-SEC. Drug Stand. Chin. 21, 429–434. 10.19778/j.chp.2020.05.011

[B12] Japan Pharmacopoeia Committee (2016). The Japanese Pharmacopoeia: XVII Seventeenth Edition ( JP17 ). Tokyo: Ministry of Health, Welfare and Drug Administration, 634–635.

[B13] Li J. Wang L.-X. Yao S.-C. Hu C. Q. (2013). Characterization of Impurities in Cefdinir Bulk Material by Online Column- Switching Liquid Chromatography and Tandem Mass Spectrometry. Curr. Pharm. Anal. 9, 145–158. 10.2174/1573412911309020004

[B14] Li J. Yao S.-C. Yin L.-H. (2021a). Analysis of Polymer Impurities in Cefmenoxime Hydrochloride Raw Materials and Preparations. Chin. J. Pharm. Anal. 41, 169–179. 10.16155/j.0254-1793.2021.01

[B15] Li J. Yao S.-C. Yin L.-H. (2021c). Analysis of Polymer Impurities in Cefoxitin Sodium for Injection. Chin. J. New Drug 30, 1038–1047. 10.3969/j.issn.1003-3734.2021.11.014

[B16] Li J. Yao S.-C. Yin L.-H. (2021b). Analysis of Polymer Impurities in Ceftazidime and Avibactam Sodium for Injection. Chin. J. New Drug 30, 1117–1125. 10.3969/j.issn.1003-3734.2021.12.011

[B17] Li J. Yao S.-C. Yin L.-H. (2021d). Characterization of Cephalosporin E-Isomer Impurities by NMR. Chin. Pharm. J. 56, 836–841. 10.11669/cpj.2021.10.011

[B18] Li J. Yao S.-C. Yin L.-H. (2020). Polymer Impurity Analysis of Cefotaxime Sodium Raw Material. Chin. J. Antibio 45, 883–892. 10.3969/j.issn.1001-8689.2020.09.008

[B19] Qian H. Wang Y.-Y. Luo Y. (2017). Analysis of Polymer Impurities in Azlocillin Sodium for Injection by Two-Dimensional High Performance Gel Chromatography with Reversed-phase Liquid Chromatography-Mass Spectrometry. Chin. Pharm. J. 52, 1273–1279. 10.11669/cpj.2017.14.017

[B20] Sha Yi. Zhang P. Li N. (2007). Nuclear Magnetic Resonance Spectroscopy of Cephalosporins. J. Spectrosc. 3, 347–352. 10.3969/j.issn.1000-4556.2007.03.015

[B21] United States Pharmacopeial Convention Inc (2020). The United States Pharmacopeia. 42 Ed. Roehville MD: the United States Pharmacopeial Convention Inc, 832–834.

[B22] Wang J. Zhou J. Xu Y. (2019). Study of the Impurity Profile and Polymerized Impurity in Mezlocillin Sodium by Multiple Heart-Cutting Two-Dimensional Liquid Chromatography Coupled with Ion Trap Time-Of-Flight Mass Spectrometry. Rapid Commun. Mass. Spectrom. 33, 1410–1419. 10.1002/rcm.8486 31148276

[B23] Wang Y.-Y. Chen Y. Hong L.-Y. (2015). Progress in the Separation and Detection of Polymer Impurities in β-lactam Antibiotics. Chin. Pharm. Aff. 29, 608–612. 10.16153/j.1002-7777.2015.06.009

[B24] Xu Y. Wang D.-D. Zhu B.-Q. (2018). Separation and Characterization of Allergenic Polymerized Impurities from Cephalosporin for Injection by Trap Free Two-Dimensional High Performance Size Exclusion Chromatography × Reversed Phase Liquid Chromatography Coupled with Ion Trap Time-Of-Flight Mass Spectrometry – ScienceDirect. J. Pharmaceut Biomed. 154, 425–432. 10.1016/j.saa.2017.11.058 10.1016/j.saa.2017.11.058 29579634

[B25] Zhang X. Li J. Wang C. (2021). Study on Polymer Impurities in Cefazolin Sodium Raw Materials and Preparations. J. Pharm. 56, 1677–1682. 10.16438/j.0513-4870.2020-1601

