# Supplementary material for: Method Improving for Isolation and Characterization of Allergy-Inducing Polymer Impurities in Cefotaxime Sodium Medicines Available From Pharmaceutical Industry
Source: Front Chem. 2022 Feb 28;10:820730. doi: 10.3389/fchem.2022.820730 (PMC8918496; doi:10.3389/fchem.2022.820730)
Supplement: Supplementary file 1 [file DataSheet1.docx]

Supplemental data of cefotaxime dimer and trimer structural characterization


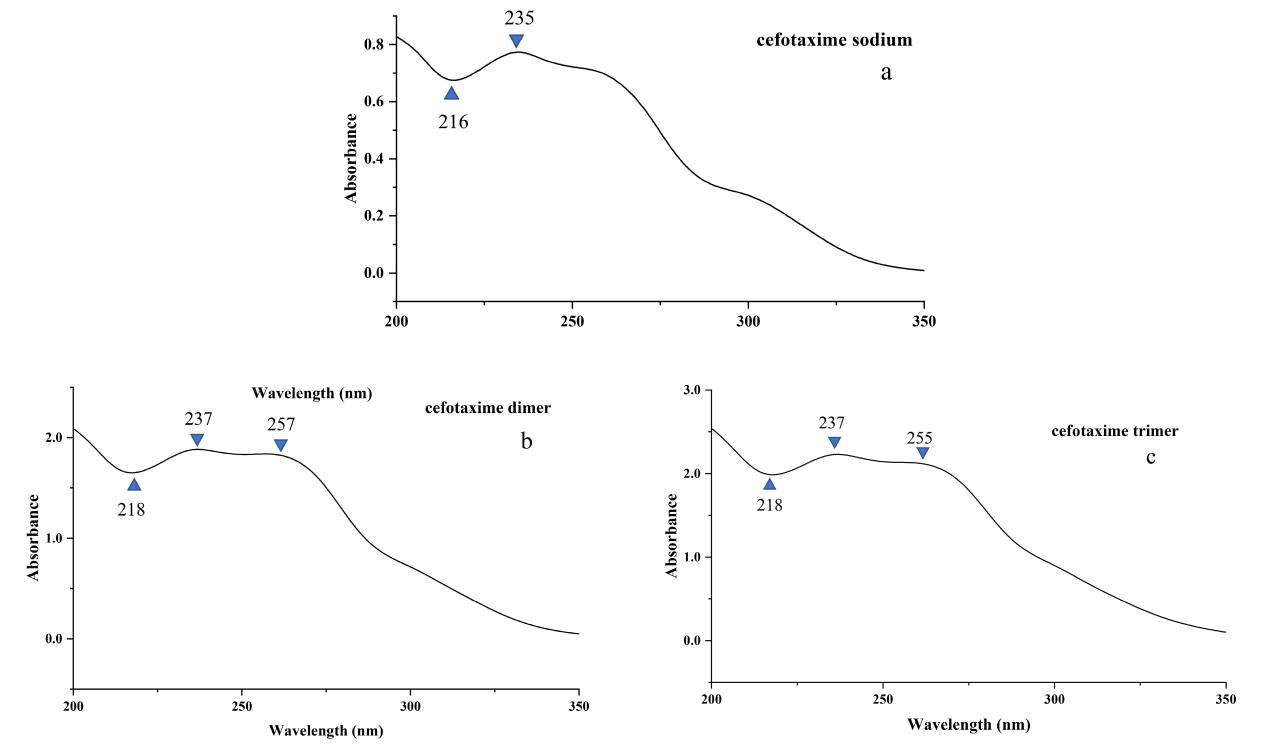


a. cefotaxime sodium；b. cefotaxime dimer；c.cefotaxime trimer

Fig.S1 UV spectra of cefotaxime sodium, cefotaxime dimer and cefotaxime trimer


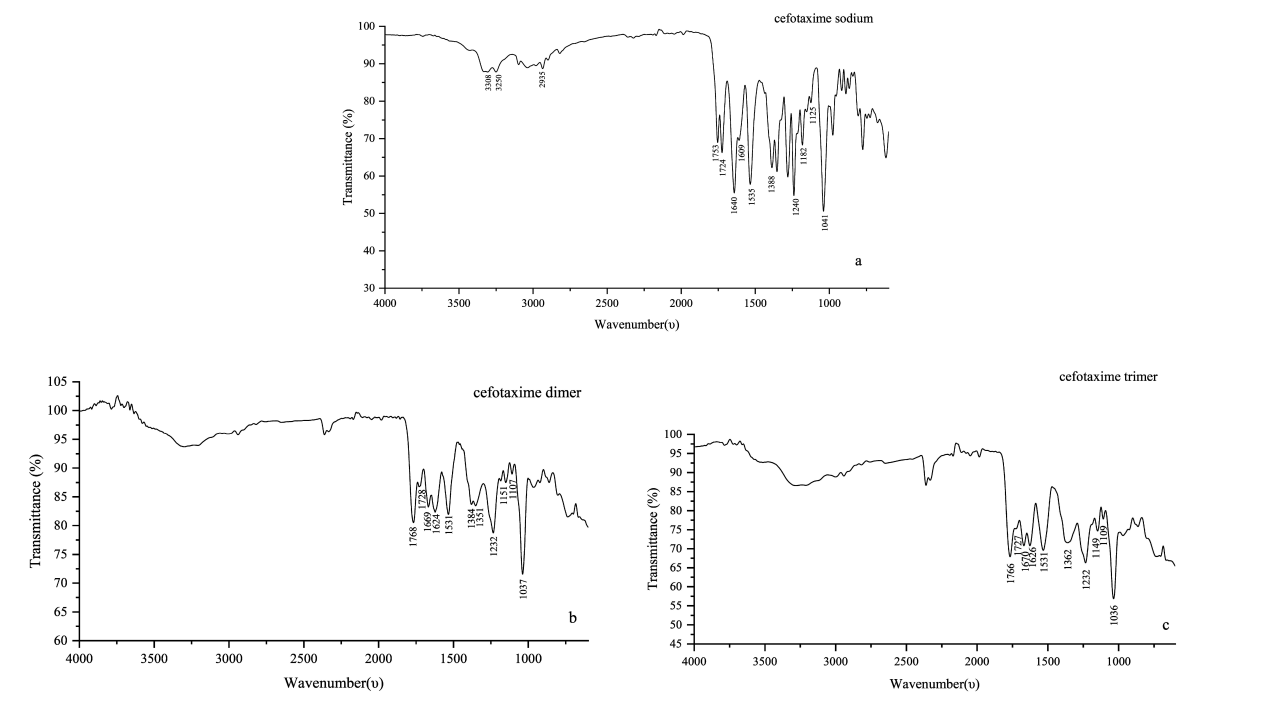


a. cefotaxime sodium；b. cefotaxime dimer；c. cefotaxime trimer

Fig.S2 IR spectra of cefotaxime sodium、cefotaxime dimer and cefotaxime trimer


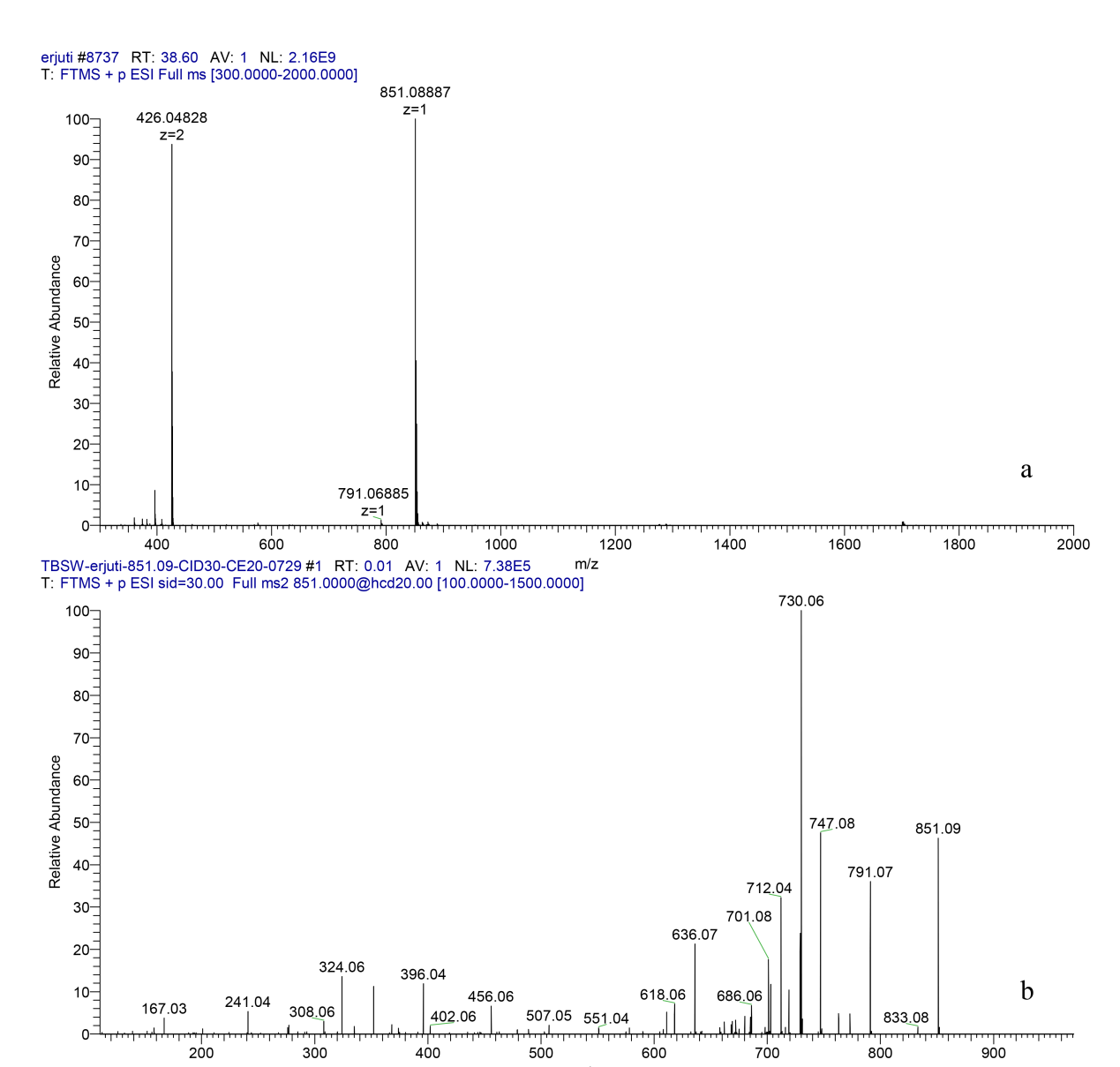


a.Full mass spectrum；[b. second](mailto:b. second) mass spectrum@851.09[M+H]^+^

Fig.S3 Typical MS spectra of cefotaxime dimer


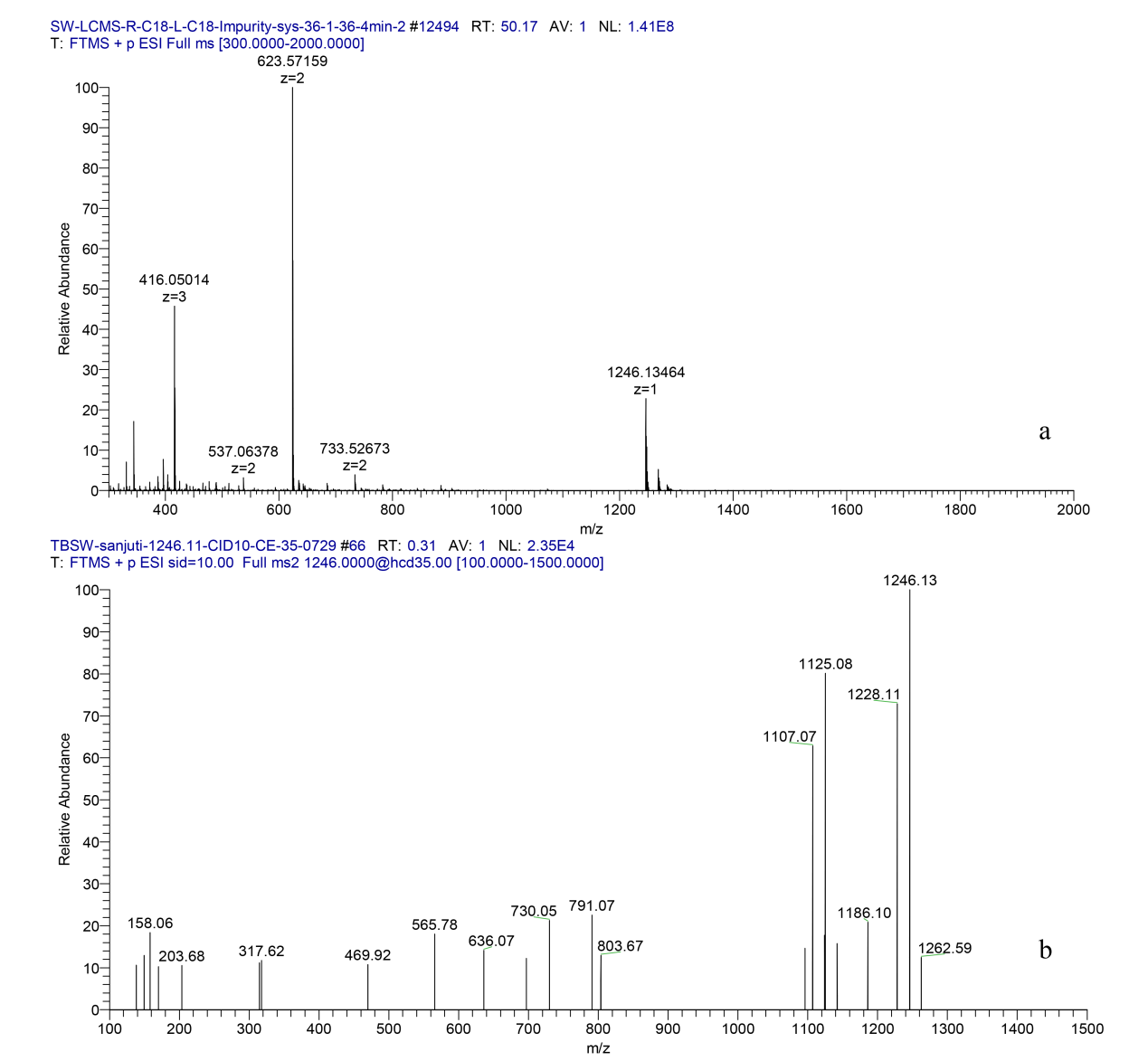


a.Full mass spectrum；[b. second](mailto:b.%20second) mass spectrum@1246.13[M+H]^+^

Fig.S4 Typical MS spectra of cefotaxime trimer


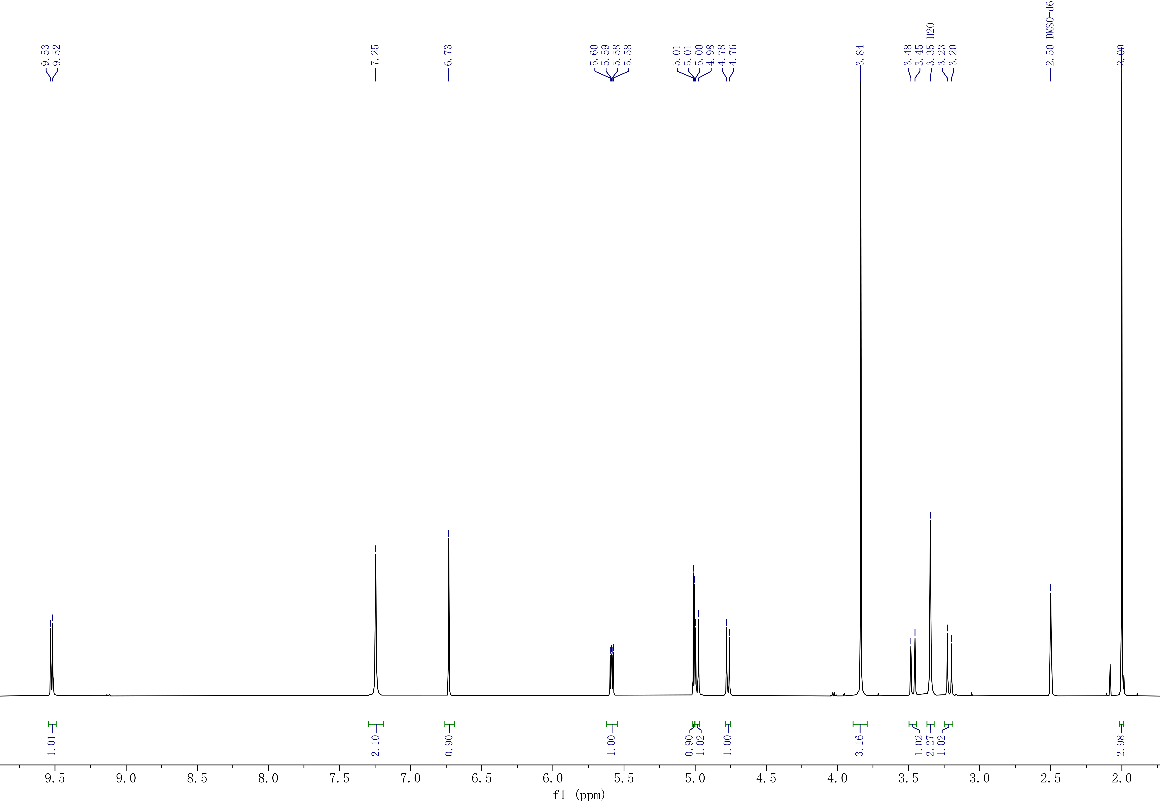


Fig.S5 The ^1^H-NMR spectrum of cefotaxime sodium


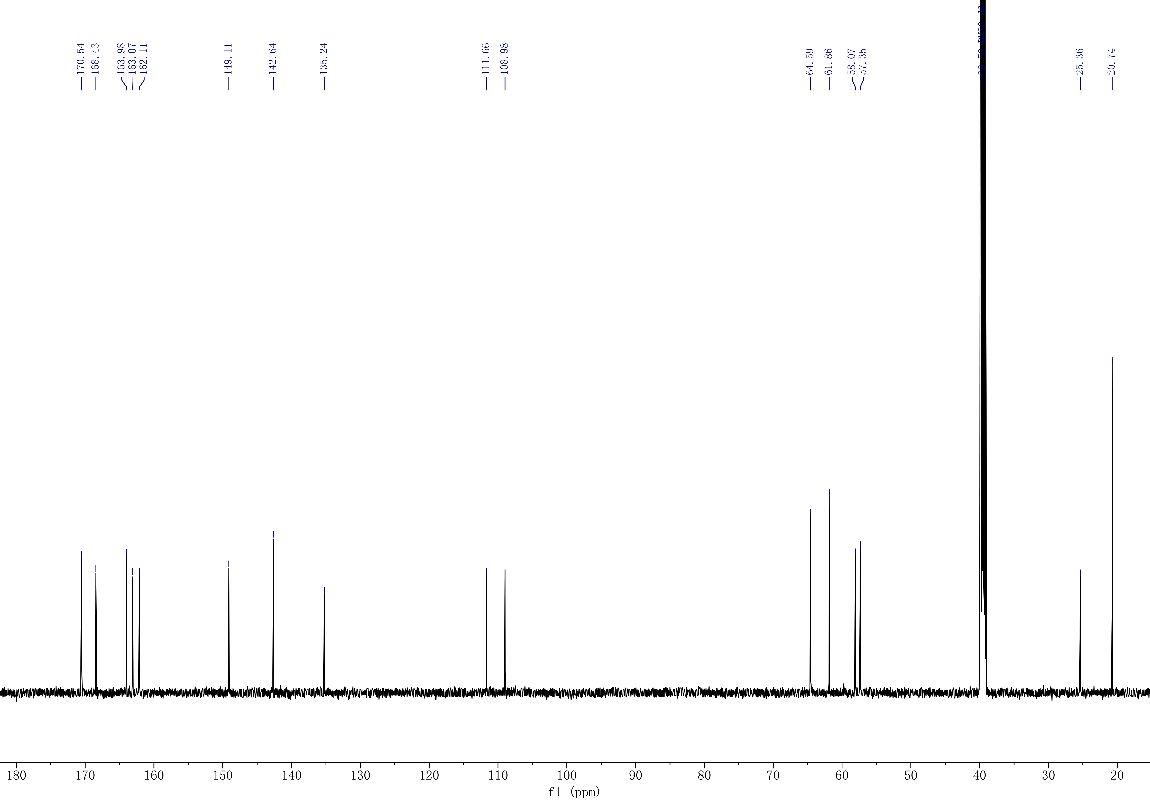


Fig.S6 The ^13^C-NMR spectrum of cefotaxime sodium


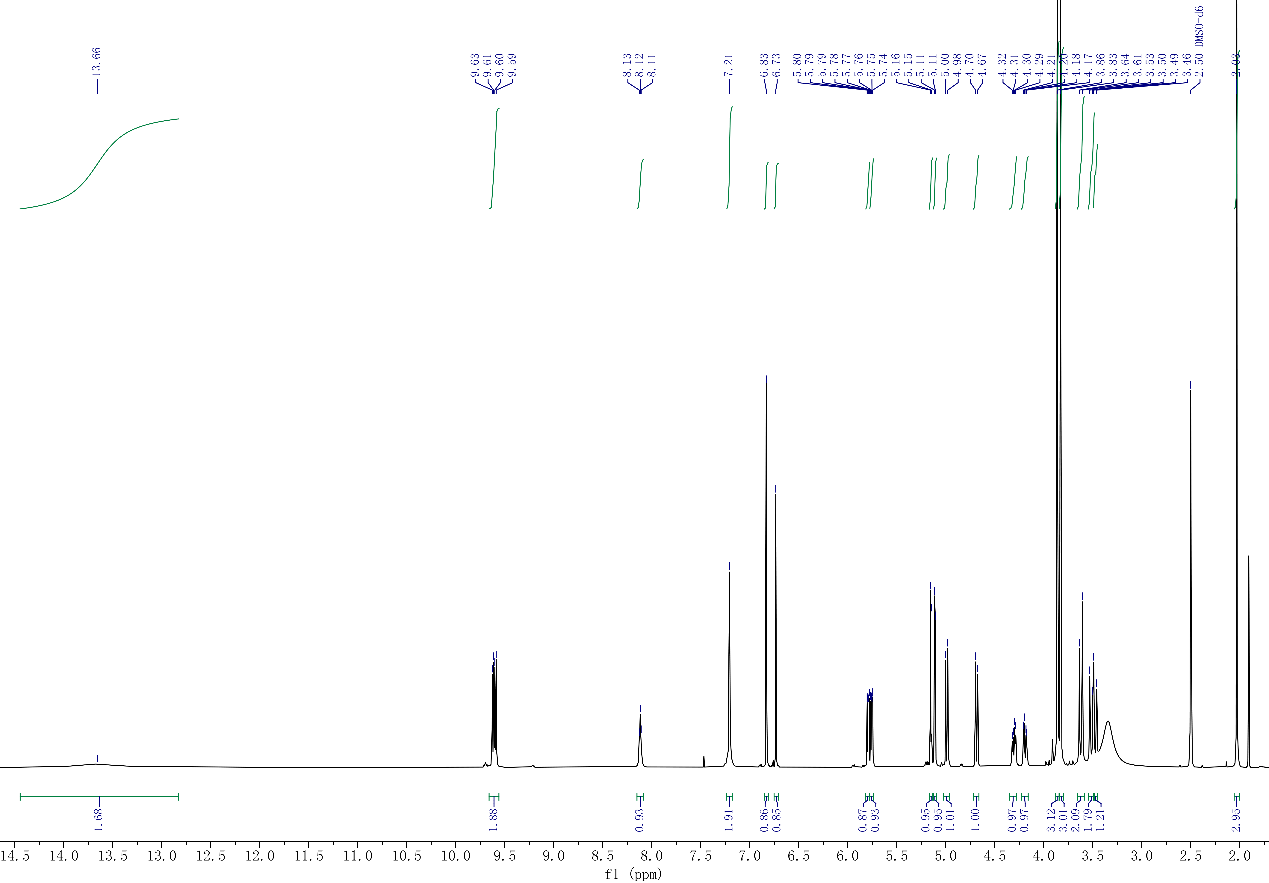
Fig.S7 The ^1^H-NMR spectrum of cefotaxime dimer


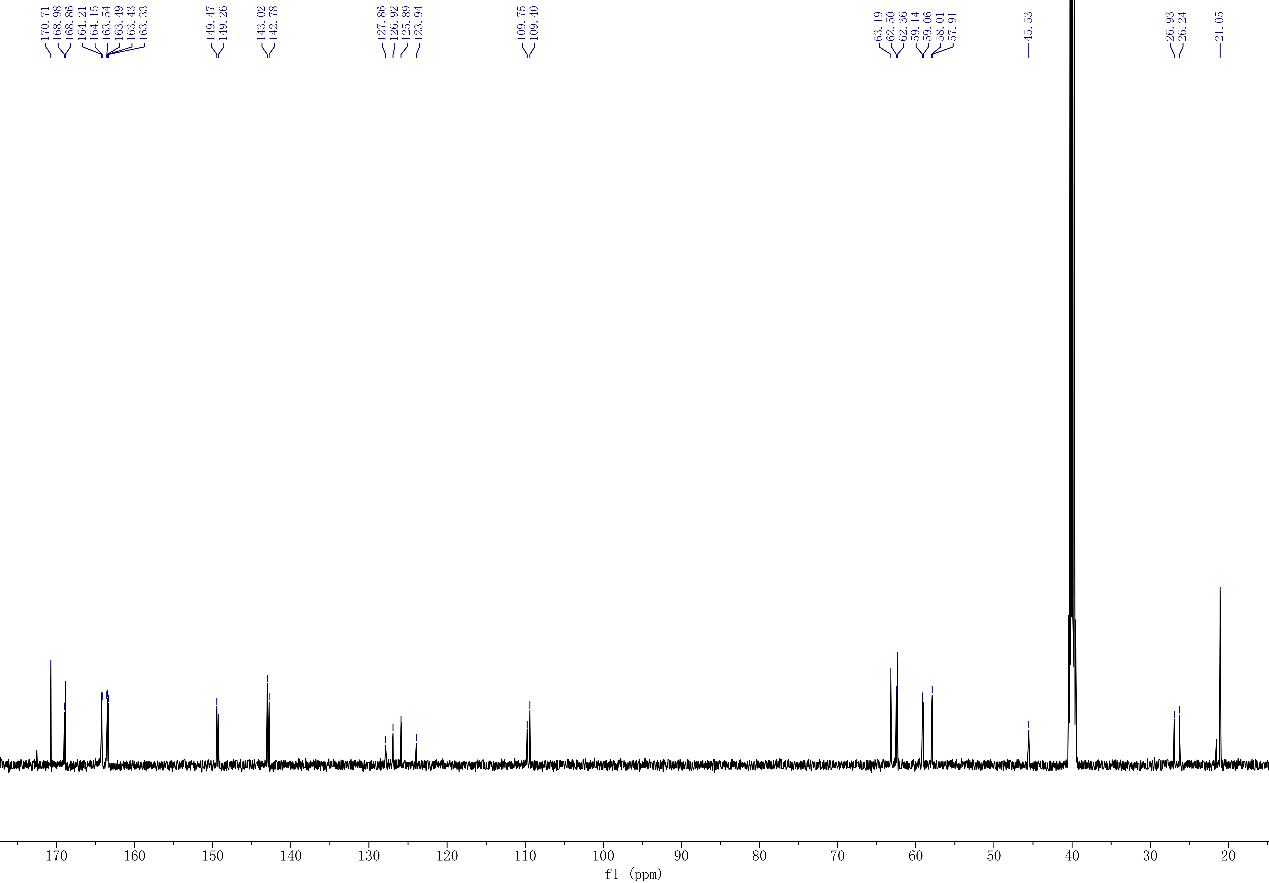
Fig.S8 The ^13^C-NMR spectrum of cefotaxime dimer


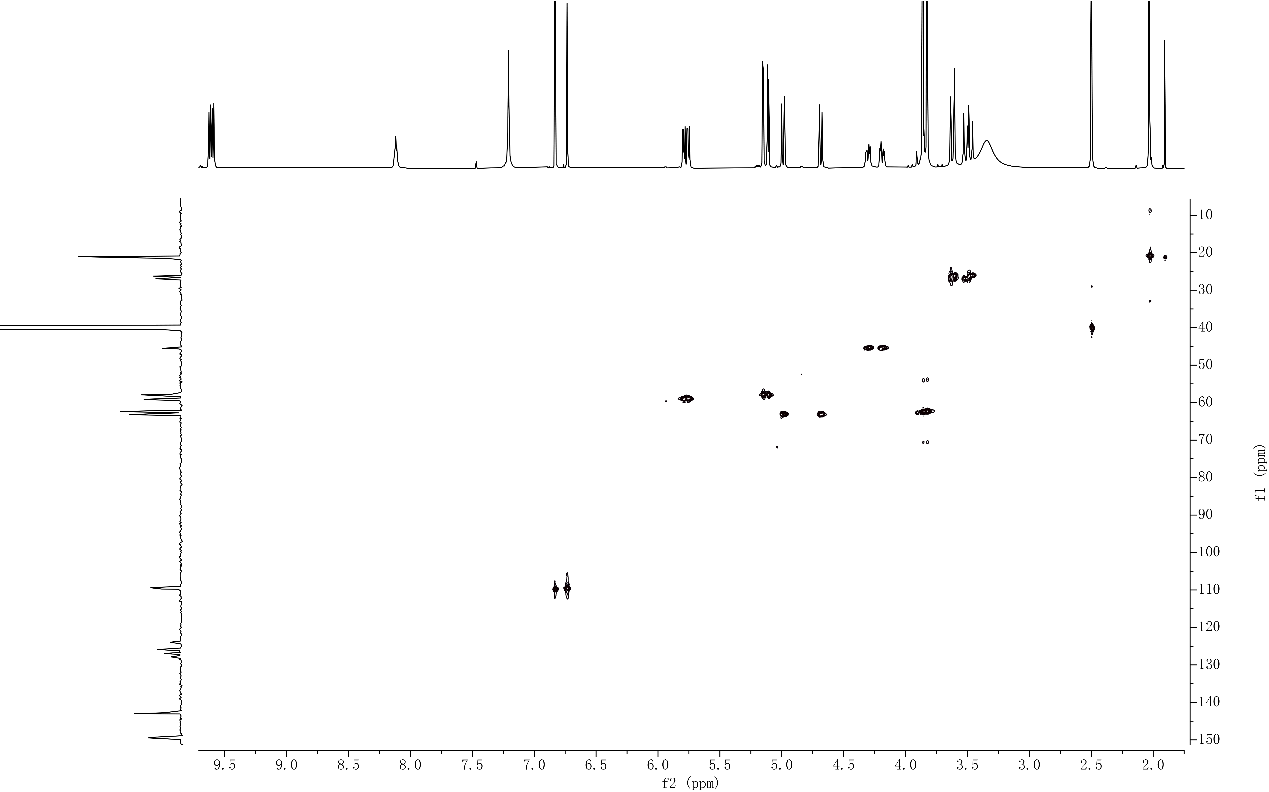
Fig.S9 The HSQC spectrum of cefotaxime dimer


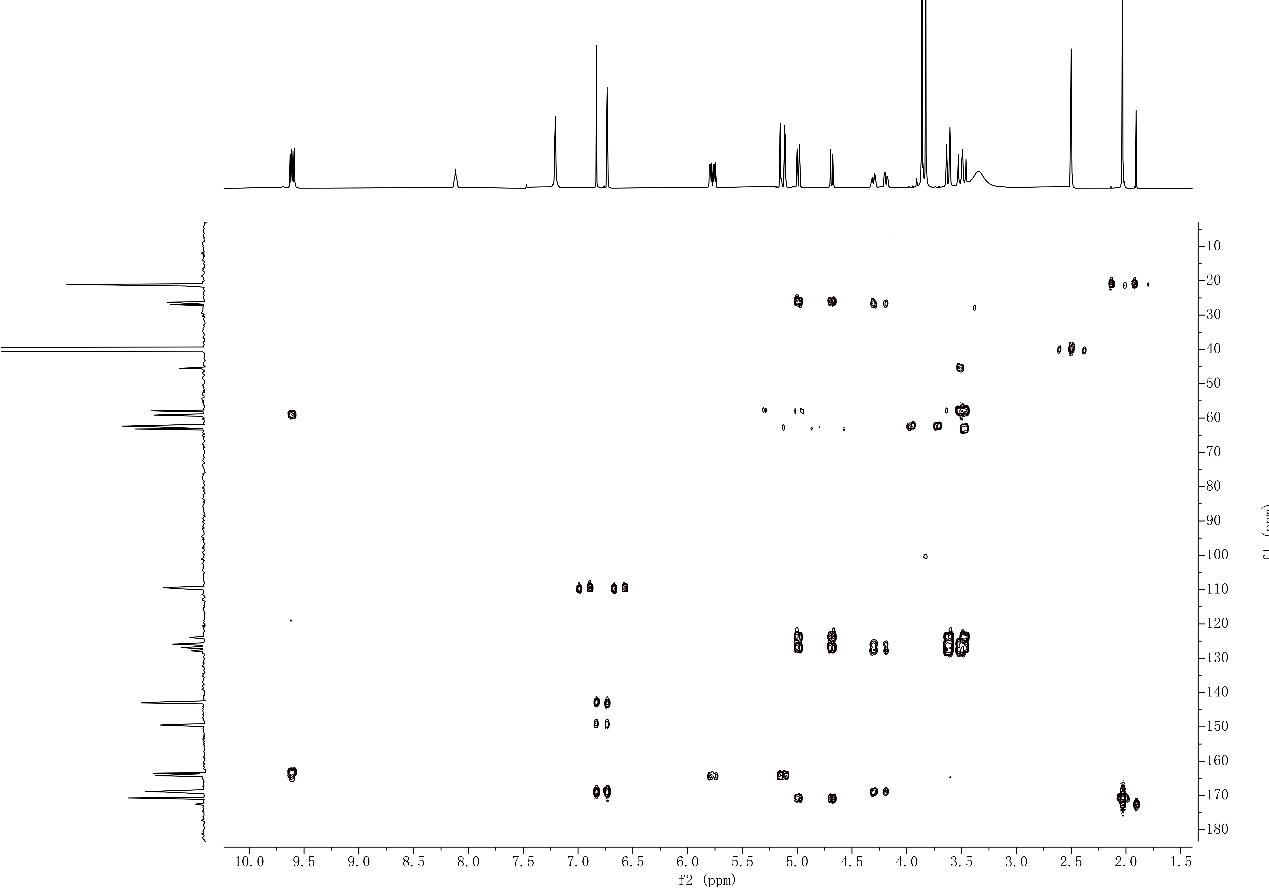

Fig.S10 The HMBC spectrum of cefotaxime dimer


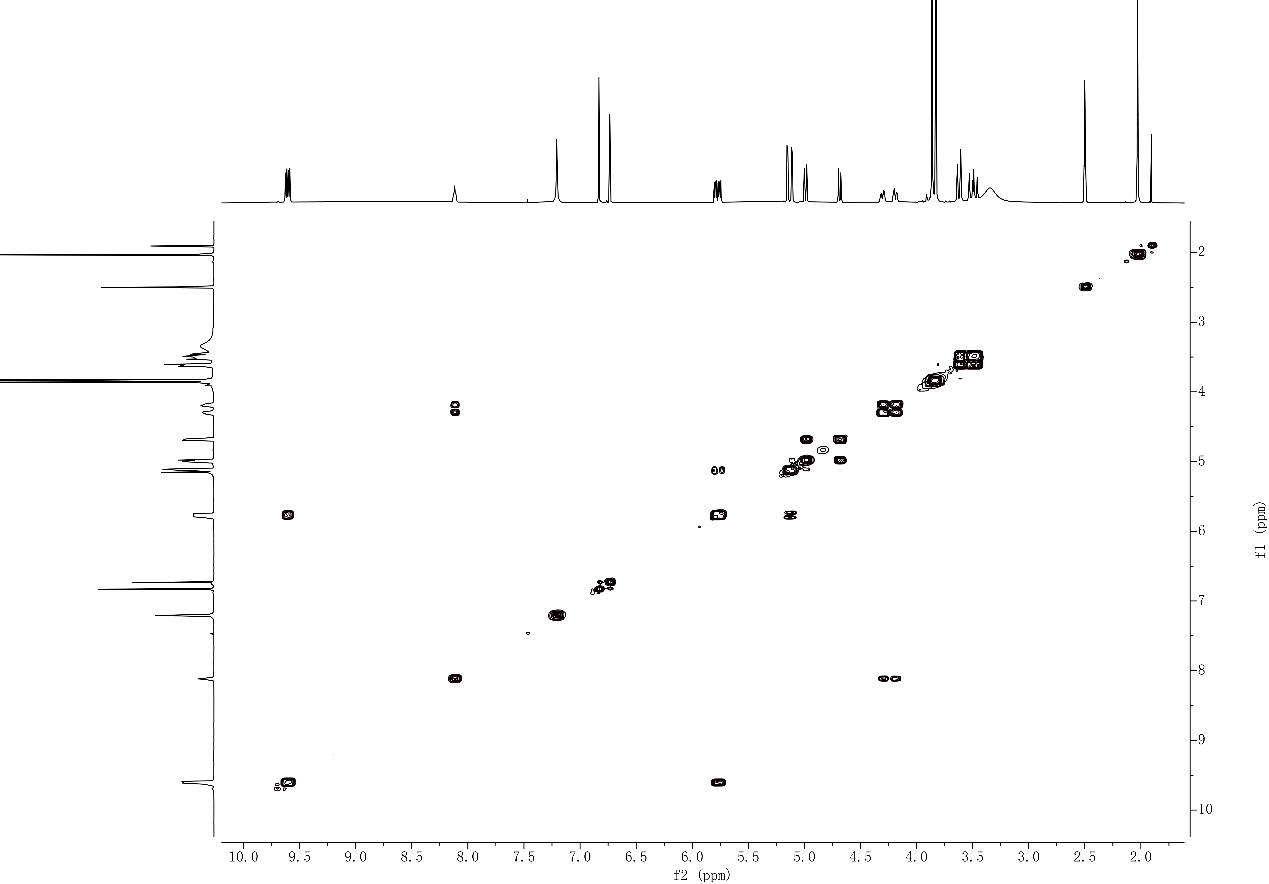
Fig.S11 The ^1^H-^1^H COSY spectrum of cefotaxime dimer


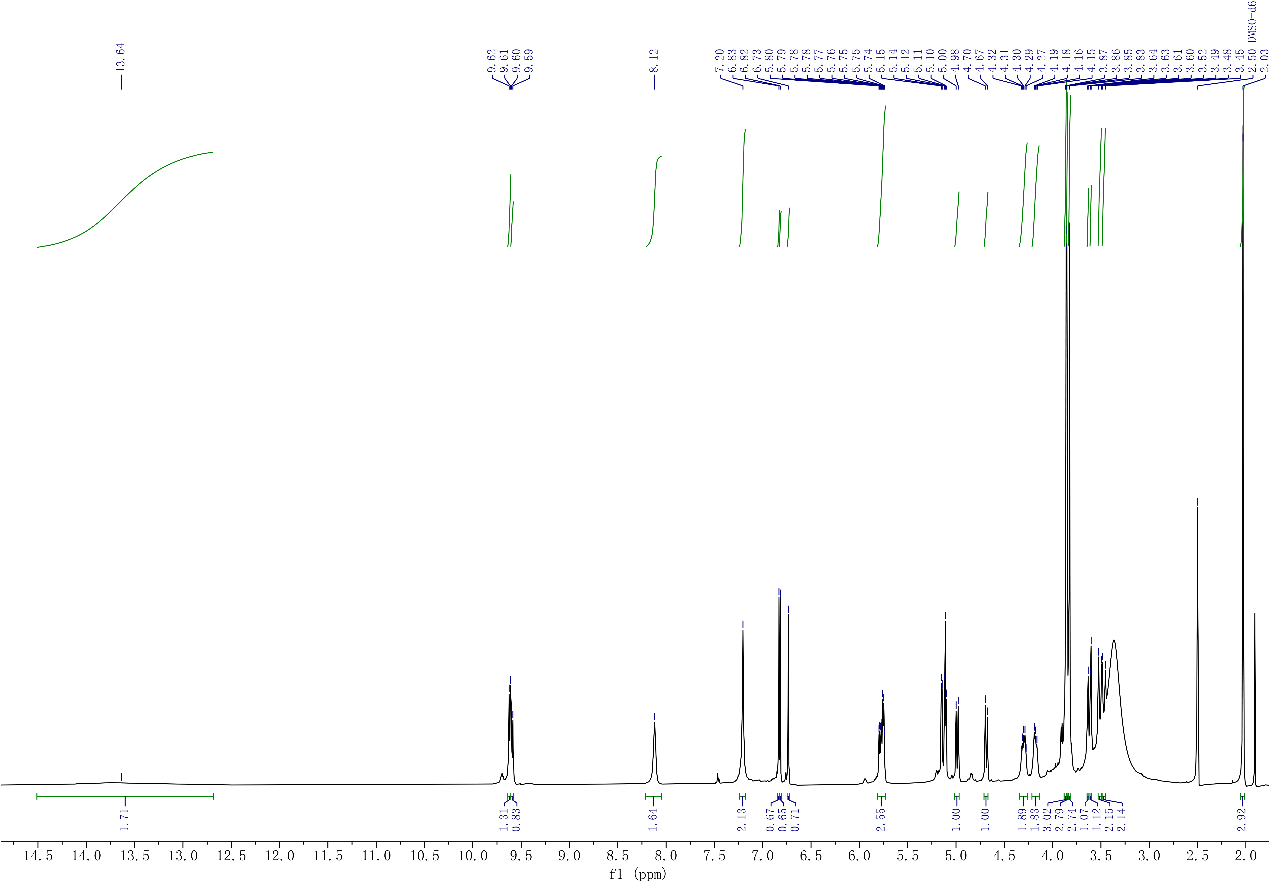


Fig.S12 The ^1^H-NMR spectrum of cefotaxime timer


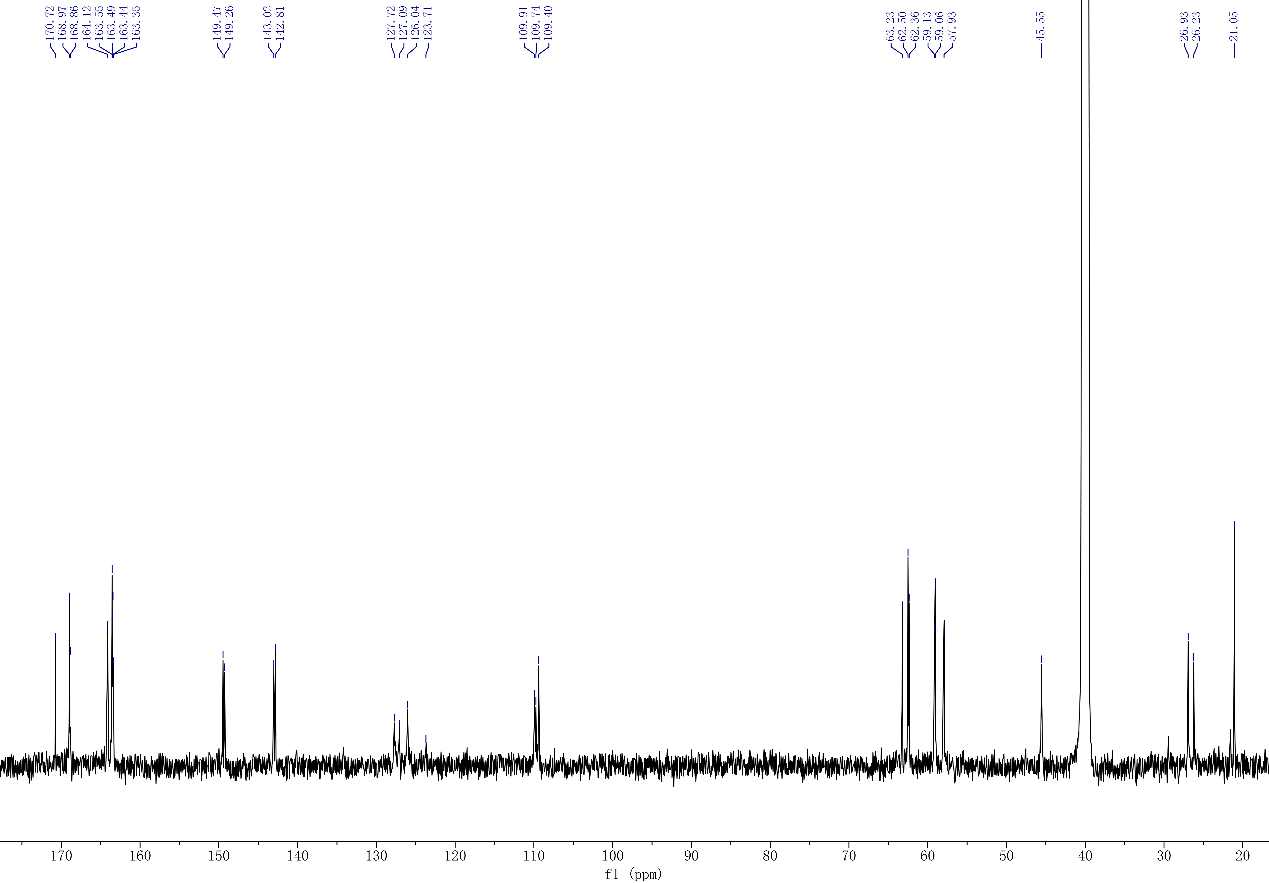


Fig.S13 The ^13^C-NMR spectrum of cefotaxime timer


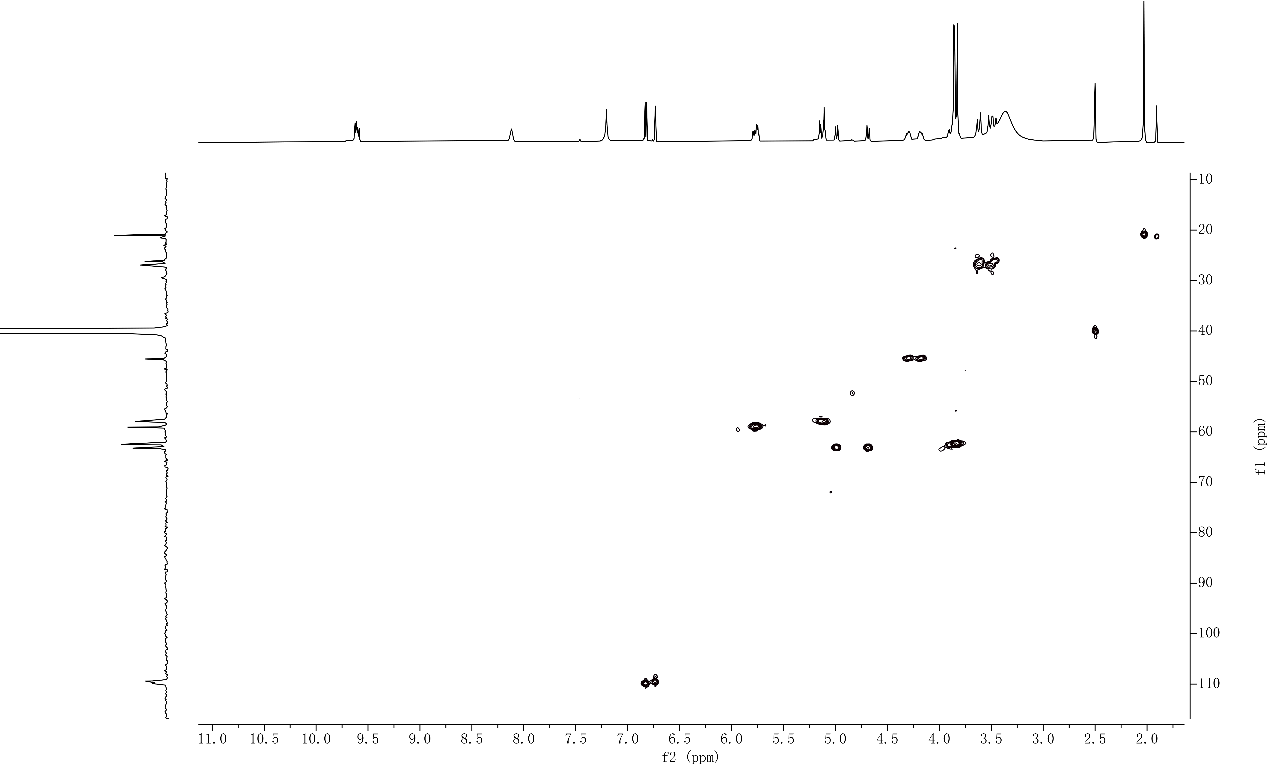


Fig.S14 The HSQC spectrum of cefotaxime timer


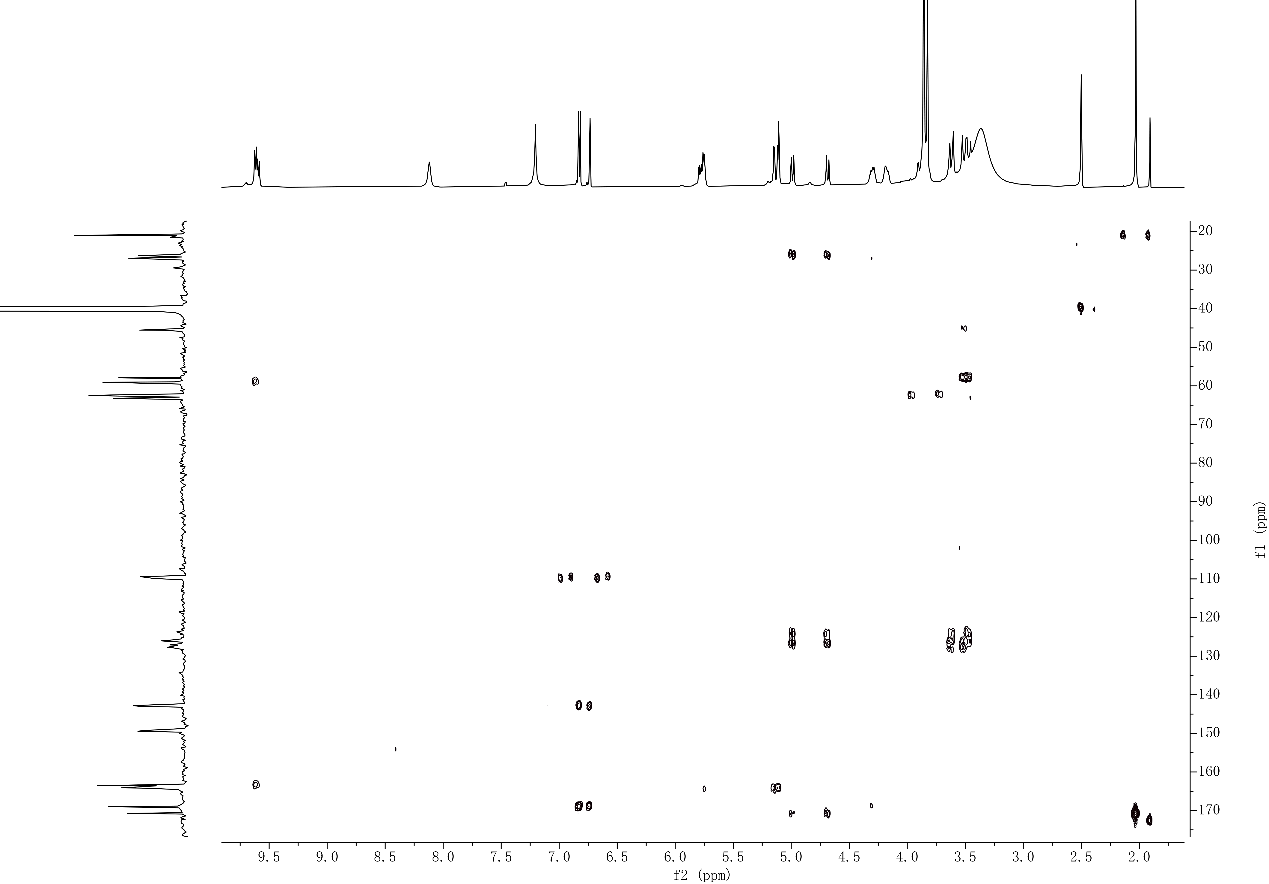

Fig.S15 The HMBC spectrum of cefotaxime timer


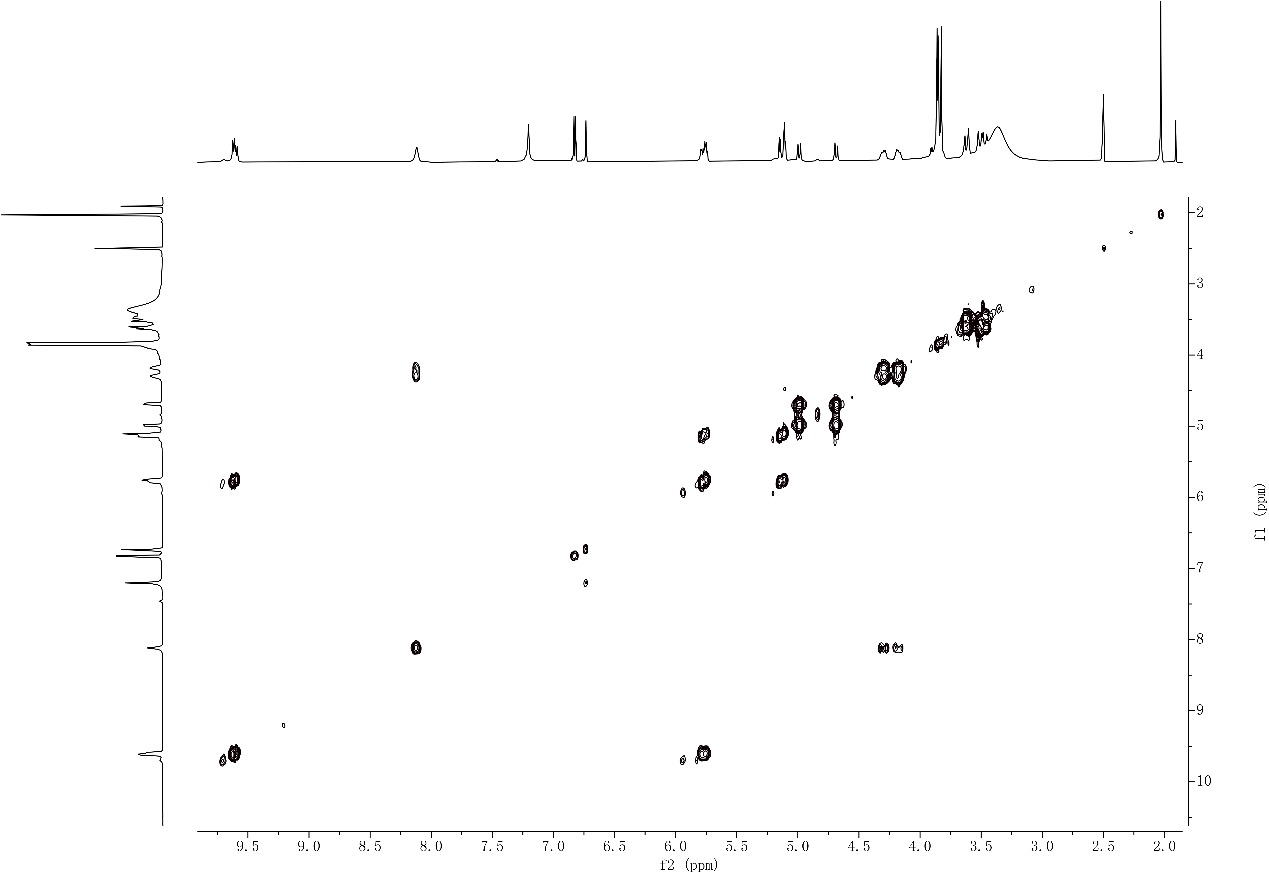


Fig.S16 The ^1^H-^1^H COSY spectrum of cefotaxime timer
